# Vascular Endothelial NAMPT‐Mediated NAD ^+^ Biosynthesis Regulates Angiogenesis and Cardiometabolic Functions in Male Mice

**DOI:** 10.1111/acel.70222

**Published:** 2025-09-29

**Authors:** Shotaro Kosugi, Shintaro Yamaguchi, Ken Nishioka, Taichi Nagahisa, Yasuhiro Watanabe, Daiki Kojima, Kenji Kaneko, Ryunosuke Mitsuno, Ran Nakamichi, Yoshinaga Kawano, Kenichiro Kinouchi, Koichiro Homma, Takeshi Kanda, Junichiro Irie, Kazutoshi Miyashita, Toshiaki Monkawa, Jun Yoshino, Hiroshi Itoh, Kaori Hayashi

**Affiliations:** ^1^ Division of Endocrinology, Metabolism and Nephrology, Department of Internal Medicine Keio University School of Medicine Tokyo Japan; ^2^ Medical Education Center Keio University School of Medicine Tokyo Japan; ^3^ Department of Emergency and Critical Care Medicine Keio University School of Medicine Tokyo Japan; ^4^ Division of Nephrology, Department of Internal Medicine Faculty of Medicine, Shimane University Izumo Japan; ^5^ Center for Integrated Kidney Research and Advance (IKRA) Faculty of Medicine, Shimane University Izumo Japan; ^6^ Division of Diabetes, Department of Medicine II Kansai Medical University Osaka Japan; ^7^ Department of Internal Medicine International University of Health and Welfare Mita Hospital Tokyo Japan; ^8^ Center for Preventive Medicine Keio University Hospital Tokyo Japan

**Keywords:** aging, angiogenesis, glucose metabolism, hypertension, NAMPT‐mediated NAD^+^ biosynthesis, vascular endothelial cell

## Abstract

Aging is associated with metabolic dysfunction and cardiovascular abnormalities. Defective nicotinamide adenine dinucleotide (NAD^+^) biosynthesis correlates with aging and aging‐associated complications. However, the precise molecular mechanisms linking aging‐associated NAD^+^ deficiency to cardiometabolic dysfunction remain unclear. Herein, we examined whether nicotinamide phosphoribosyltransferase (NAMPT), a key enzyme in NAD^+^ biosynthesis, influences vascular endothelial function and whole‐body metabolic and hemodynamic homeostasis during aging. Vascular endothelial cell–specific *Nampt* knockout (VeNKO) mice fed a regular chow diet exhibited no cardiometabolic abnormalities, whereas male VeNKO mice fed a high‐fat diet exhibited reduced angiogenesis, resulting in impaired subcutaneous adipogenesis, impaired glucose metabolism, and hemodynamic disturbances. Mechanistically, NAMPT loss attenuated NAD^+^‐dependent deacetylase sirtuin‐1 (SIRT1) and endothelial nitric oxide synthase (eNOS) signaling, impairing angiogenesis. Aged mice exhibited endothelial NAD^+^ depletion driven by an imbalance between NAMPT‐mediated NAD^+^ biosynthesis and consumption, leading to impaired eNOS signaling and associated angiogenic and cardiometabolic dysfunction, similar to that observed in VeNKO mice. Nicotinamide mononucleotide administration replenished vascular endothelial NAD^+^ levels, improved angiogenesis, restored subcutaneous adipose tissue volume, and ameliorated aging‐associated cardiometabolic dysfunction. Collectively, our findings provide mechanistic and therapeutic insights into vascular endothelial NAMPT–NAD^+^–SIRT1–eNOS signaling related to aging‐associated cardiometabolic disorders.

## Graphical abstract

Using male vascular endothelial cell–specific *Nampt* knockout and naturally aged mice, we identified endothelial NAD^+^ deficiency as a central driver of age‐related angiogenic failure, impaired subcutaneous adipogenesis, and cardiometabolic dysfunction. NAD^+^ deficiency disrupts SIRT1‐dependent eNOS regulation by reducing Ser‐1177 phosphorylation and increasing acetylation, thereby attenuating angiogenesis. Restoring endothelial NAD^+^ levels with nicotinamide mononucleotide improves angiogenesis, subcutaneous adipose tissue volume, and cardiometabolic function, highlighting the NAMPT–NAD^+^–SIRT1–eNOS axis as a mechanistic and therapeutic target in aging‐associated cardiometabolic diseases.

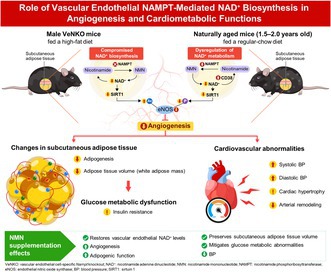

## 1 Introduction

Aging is associated with impaired glucose metabolism, decreased daily energy expenditure, and multiple cardiovascular abnormalities, including hypertension, increased arterial intima‐media thickness, and cardiac hypertrophy (Hwang et al. 2022; Palmer and Jensen 2022). The mechanisms underlying aging‐induced cardiometabolic changes remain unclear but likely involve endothelial dysfunction, which affects oxygen and nutrient supply, blood flow regulation, vascular tone, and angiogenesis (Hwang et al. 2022; Palmer and Jensen 2022). Aging impairs angiogenesis, which is closely linked to adipogenesis and energy expenditure, thereby further regulating whole‐body glucose and energy metabolism (Christiaens and Lijnen 2009; Palmer and Jensen 2022; Sung et al. 2013; Xu et al. 2012). Additionally, aging‐induced angiogenesis impairment likely contributes to high blood pressure (BP) and cardiovascular diseases (Hwang et al. 2022). However, the precise molecular mechanisms linking aging‐associated angiogenic dysfunction to these cardiometabolic disorders remain unclear.

Nicotinamide adenine dinucleotide (NAD^+^) is a critical coenzyme in metabolic redox reactions and an essential substrate for multiple NAD^+^‐consuming enzymes, including members of the sirtuin (Sirt) family (Chini et al. 2023, 2021; Nagahisa et al. 2023). These enzymes play vital roles in metabolic homeostasis, DNA repair, stress resistance, circadian rhythm regulation, behavior, and cognition (Chini et al. 2023, 2021; Nagahisa et al. 2023). The regulation of cellular NAD^+^ levels involves multiple enzymes that participate in NAD^+^ biosynthesis, including nicotinamide phosphoribosyltransferase (NAMPT), a rate‐limiting enzyme in mammals, nicotinamide mononucleotide adenylyltransferases, and NAD^+^‐consuming enzymes such as CD38 and poly (ADP‐ribose) polymerases (Chini et al. 2023, 2021; Nagahisa et al. 2023).

Impaired NAMPT‐mediated NAD^+^ biosynthesis contributes to various aging‐associated metabolic diseases, including disrupted energy metabolism, beta‐cell dysfunction, insufficient glucagon‐like peptide‐1 production, glucose intolerance, insulin resistance, and type 2 diabetes (Chini et al. 2023, 2021; Nagahisa et al. 2023; Yamaguchi et al. 2019). Furthermore, NAMPT expression was reduced in the aortic wall tissues of patients with hypertension and rats with angiotensin II (Ang‐II)‐induced hypertension, accompanied by aortic wall fibrosis. NAMPT haplodeficiency exacerbates Ang‐II‐induced hypertension (Zhou et al. 2020). Notably, NAMPT‐mediated NAD^+^ biosynthesis in vascular endothelial cells is compromised in replicative aging, inhibiting angiogenesis in vitro (Borradaile and Pickering 2009). Endothelial cells obtained from aged mice exhibit NAD^+^ depletion, which suppresses ATP production by inhibiting NAD^+^‐dependent enzymes in glycolysis and the TCA cycle, limiting energy availability for angiogenesis (Kiesworo et al. 2024). These findings have indicated the interplay between vascular NAD^+^ biosynthesis, angiogenesis, and aging‐associated metabolic and hemodynamic disorders. However, the role of vascular endothelial NAD^+^ metabolism in aging‐associated cardiometabolic dysfunction remains poorly understood.

We hypothesized that vascular endothelial NAMPT‐mediated NAD^+^ biosynthesis is essential for angiogenesis regulation and that its impairment contributes to the development of aging‐associated abnormalities in glucose and energy metabolism, as well as hemodynamic homeostasis. To test this hypothesis, we generated vascular endothelial cell‐specific *Nampt* knockout (VeNKO) mice and examined their angiogenic function, whole‐body glucose and energy metabolism, and circulatory phenotypes. In addition, we evaluated changes in vascular endothelial NAD^+^ metabolism in naturally aged mice and further explored the therapeutic potential of promoting vascular endothelial NAD^+^ biology to counteract aging‐associated cardiometabolic disorders.

## 2 Results

### 2.1 Establishment of VeNKO Mice

**FIGURE 1:**
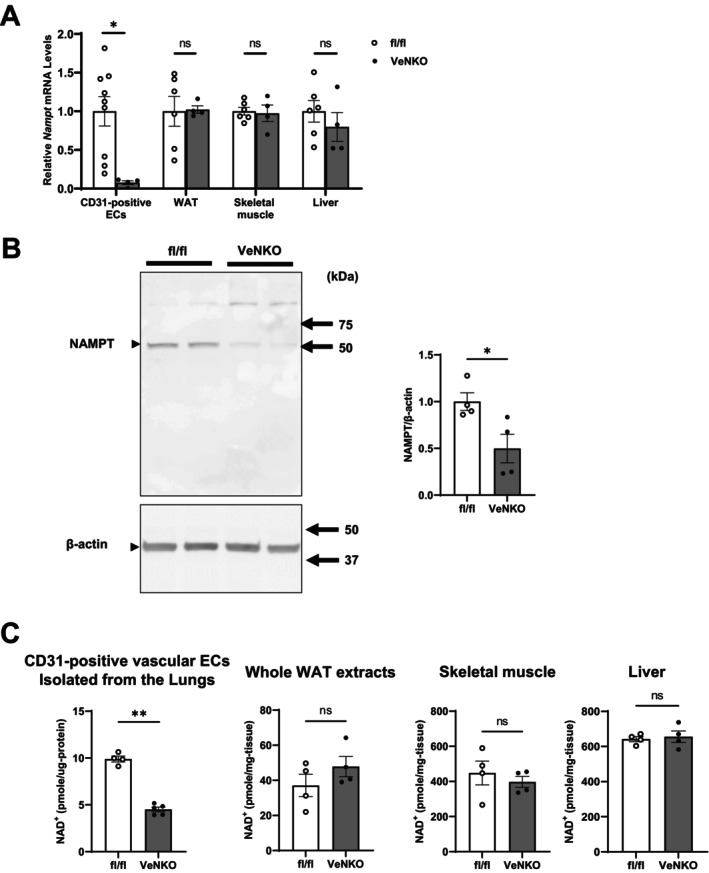
Generation of vascular endothelial cell–specific *Nampt* knockout (VeNKO) mice. VeNKO mice were generated by crossing floxed‐*Nampt* (fl/fl) mice with transgenic mice expressing the *Cre* recombinase under the control of the *Ve‐cadherin* gene promoter. (A) *Nampt* expression in CD31‐positive vascular endothelial cells (ECs) isolated from the white adipose tissue (WAT), whole WAT extracts, skeletal muscle, and liver of fl/fl and VeNKO mice aged 2–6 months (*n* = 4–9 per group). (B) Western blotting and quantification of nicotinamide phosphoribosyltransferase (NAMPT) and β‐actin in CD31‐positive vascular ECs isolated from the lungs of 2–3‐month‐old fl/fl and VeNKO mice (*n* = 4 each group). (C) NAD^+^ levels in CD31‐positive vascular ECs isolated from the lungs, whole WAT extracts, skeletal muscle, and liver of 2–6‐month‐old fl/fl and VeNKO mice (*n* = 4–5 per group). Data were analyzed using Student's unpaired *t*‐test. All values are presented as the mean ± standard error of the mean (SEM). *p < 0.05, **p < 0.01.

We generated VeNKO mice by crossing mice harboring *loxP* sites flanking *Nampt* (fl/fl) with transgenic mice expressing *Cre* recombinase under the control of the *Ve‐cadherin* promoter. VeNKO mice were born following expected Mendelian ratios and appeared phenotypically normal. Vascular endothelial *Nampt* expression was similar in fl/fl and *Ve‐cadherin*‐Cre mice (Figure S1). To confirm the specificity of *Ve‐cadherin*‐Cre–mediated recombination, we quantitatively assessed *Nampt* mRNA levels in hematopoietic lineage cells included in total bone marrow isolates obtained from fl/fl and VeNKO mice. We observed no significant differences between genotypes (Figure S2), indicating that *Cre*‐mediated recombination did not occur in hematopoietic cells under our experimental conditions. However, in VeNKO mice, *Nampt* expression was markedly reduced in CD31‐positive vascular endothelial cells but not in other metabolic organs such as white adipose tissue (WAT), skeletal muscle, and liver (Figure 1A). In particular, NAMPT expression was reduced by approximately 50% in CD31‐positive vascular endothelial cells of VeNKO mice compared with that in fl/fl controls (Figure 1B). Likewise, NAD^+^ levels decreased by 54% in vascular endothelial cells but not in the WAT, skeletal muscle, or liver of VeNKO mice compared with those in fl/fl controls (Figure 1C), suggesting that NAMPT is an important regulator of NAD^+^ biosynthesis in vascular endothelial cells.

### 2.2 Male VeNKO Mice Fed a High‐Fat Diet Exhibit Insufficient Angiogenic and Adipogenic Capacities as Well as Disrupted Glucose and Hemodynamic Homeostasis

We first characterized the metabolic and hemodynamic phenotypes in male and female VeNKO and fl/fl mice fed a regular chow diet (RCD). Notably, vascular endothelial cell‐specific NAMPT deletion did not affect the body weight, fat mass, lean mass, or organ weights of mice (Figure S3A,B). In addition, we did not observe any difference in daily food intake between the two groups (Figure S3C). The loss of vascular endothelial *Nampt* did not affect the respiratory quotient or energy expenditure (Figure S3D). VeNKO mice had normal glucose and insulin tolerance (Figure S3E,F). Systolic BP (SBP) values and cardiac weights were also similar between VeNKO and fl/fl mice (Figure S3G,H).

**FIGURE 2:**
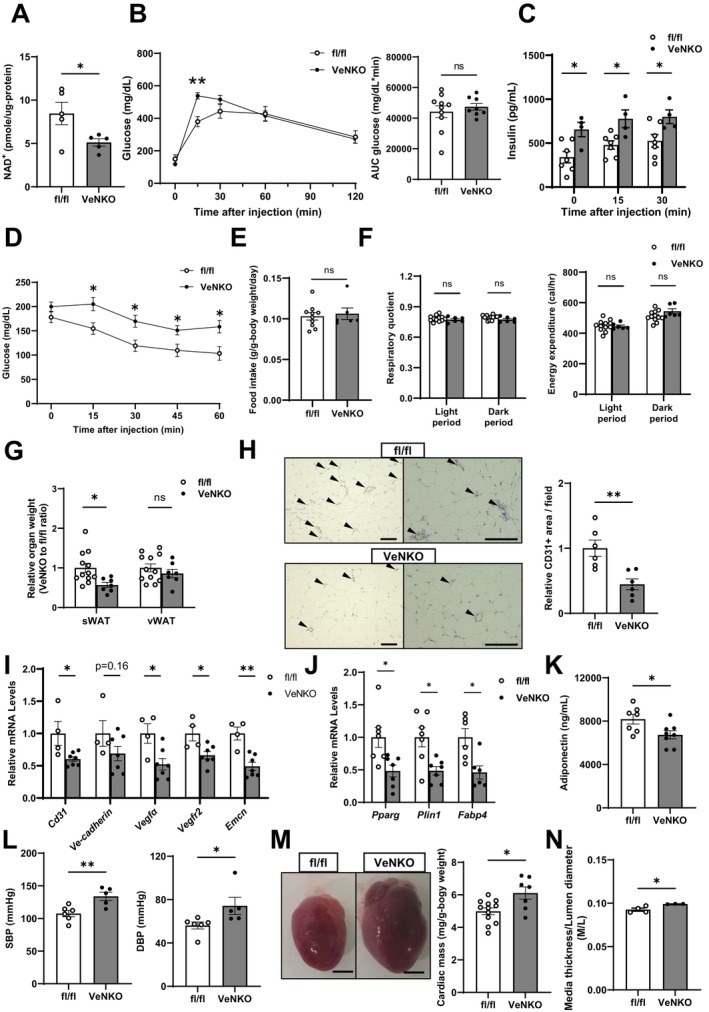
NAMPT loss impairs metabolic and cardiovascular homeostasis by modulating angiogenesis in male mice fed a high‐fat diet (HFD). Characterization of male fl/fl and VeNKO mice fed an HFD. (A) NAD^+^ levels in CD31‐positive ECs isolated from the lungs of mice after 17–19 weeks of HFD feeding (*n* = 5 per group). (B) Intraperitoneal glucose tolerance tests (IPGTTs) were performed on male VeNKO and fl/fl mice after 10–15 weeks of HFD feeding (*n* = 8–10 per group). The area under the curve (AUC) for glucose during the IPGTTs is displayed next to the glucose curves. (C) Plasma insulin concentrations during the IPGTTs following overnight fasting in male VeNKO and fl/fl mice after 10–15 weeks of HFD feeding (*n* = 4–7 per group). (D) Intraperitoneal insulin tolerance tests (ITTs) were performed on male VeNKO and fl/fl mice after 10–15 weeks of HFD feeding (*n* = 7–12 per group). (E) Daily food intake in male VeNKO and fl/fl mice after 7–10 weeks of HFD feeding (*n* = 6–10 per group). (F) Respiratory quotient, and energy expenditure (*n* = 6–12 per group) in male VeNKO and fl/fl mice after 11–14 weeks of HFD feeding. (G) Evaluation of fat depot masses, including subcutaneous white adipose tissue (sWAT) and visceral white adipose tissue (vWAT), after 15–19 weeks of HFD feeding. Relative fat mass was normalized to that of the fl/fl group, which was set as 1 (*n* = 7–12 per group). (H) Representative micrographs of immunohistochemical staining for CD31 in sWAT after 15–19 weeks of HFD feeding. Left: Low magnification; Right: High magnification. Arrowheads denote CD31‐positive cells (brown, EC marker). Scale bar, 100 μm. Quantification of CD31‐positive areas per field per mouse (*n* = 6 per group). (I) mRNA expression levels of genes involved in angiogenesis in sWAT after 15–19 weeks of HFD feeding (*n* = 4–7 per group). (J) mRNA expression levels of genes involved in adipocyte differentiation in sWAT (*n* = 6–7 per group) and (K) plasma adiponectin concentrations determined after 13–19 weeks of HFD feeding (*n* = 7–8 per group). (L) Systolic (SBP) and diastolic blood pressure (DBP) (mmHg) after 14–18 weeks of HFD feeding (*n* = 5–6 per group). (M) Representative images of the heart (scale bar, 2.5 mm) and cardiac masses after 15–19 weeks of HFD feeding (*n* = 7–12 per group). (N) Ratios of aortic media thickness to lumen diameter after 15–19 weeks of HFD feeding (*n* = 3–4 per group). Data were analyzed using Student's unpaired *t*‐test. All values are presented as the mean ± SEM. *p < 0.05, **p < 0.01.

A high‐fat diet (HFD) accelerates several aspects of biological aging in vascular endothelial cells (Lesniewski et al. 2013). Therefore, to investigate the pathophysiological roles of vascular endothelial NAD^+^ in aging‐associated energy and glucose metabolism and in circulatory diseases, VeNKO and fl/fl mice were fed an HFD. NAD^+^ levels were significantly reduced in vascular endothelial cells of male HFD‐fed VeNKO mice, similar to those in RCD‐fed mice (Figure 2A). Initially, we assessed whole‐body glucose metabolism in HFD‐fed mice using intraperitoneal glucose tolerance tests (IPGTTs) and insulin tolerance tests (ITTs). In IPGTTs, although male VeNKO mice exhibited significantly higher glucose levels at 15 min compared with fl/fl controls, overall glucose excursion assessed using the area under the curve was comparable between fl/fl and VeNKO mice (Figure 2B). To assess potential compensatory responses, we measured plasma insulin concentrations during IPGTTs and found them to be elevated in male VeNKO mice compared with fl/fl controls (Figure 2C). In ITTs, insulin injection failed to decrease blood glucose concentrations in male VeNKO mice (Figure 2D). These findings suggest that despite insulin resistance, systemic glucose tolerance was maintained, likely because of compensatory hyperinsulinemia (Stromsdorfer et al. 2016). Despite these abnormalities in whole‐body glucose metabolism, HFD‐fed male VeNKO mice had comparable total body weight, fat mass, and lean mass to fl/fl controls (Figure S4A,B). In addition, food intake, respiratory quotient, and energy expenditure were similar between VeNKO and control mice (Figure 2E,F).

Next, we examined the involvement of major metabolic organs, namely, the liver, skeletal muscle, and adipose tissue, in whole‐body glucose metabolism abnormalities. The levels of phosphorylated Akt at Ser‐473 in the liver and skeletal muscle of male VeNKO mice were comparable to those in fl/fl mice (Figure S5A,C). Consistent with this observation, the levels of hepatic and skeletal muscle triglycerides were also comparable between male VeNKO and fl/fl mice (Figure S5B,D). Based on these findings, we hypothesized that defects in vascular endothelial NAMPT‐mediated NAD^+^ biosynthesis impair the adipose tissue metabolic function, influencing whole‐body glucose metabolism. Although the triglyceride content in subcutaneous WAT (sWAT) was comparable between male VeNKO and fl/fl mice, the levels of phosphorylated Akt at Ser‐473 were reduced in sWAT from male VeNKO mice compared with those in controls (Figure S5E,F). We also found that the sWAT mass but not the visceral WAT (vWAT) mass was significantly reduced in male VeNKO mice (Figure 2G).

Reduced fat mass has been attributed to impaired angiogenesis (Xu et al. 2012); therefore, we quantified the CD31‐positive sWAT vascular endothelial cells and found that capillary density was decreased in male VeNKO mice compared with that in male fl/fl controls (Figure 2H). Consistent with this observation, we observed significantly reduced expression of angiogenesis genes such as *CD31*, *Vegfα*, *Vegfr2*, and *Emcn* and a trend toward reduced *Ve‐cadherin* expression in the sWAT of male VeNKO mice compared with that in their counterparts (Figure 2I). We next assessed the expression levels of the sWAT adipocyte differentiation genes *Pparg*, *Plin1*, and *Fabp4*, as well as the plasma concentration of the insulin‐sensitizing adipokine, adiponectin. We observed significantly decreased expression of *Pparg*, *Plin1*, and *Fabp4* in the sWAT of male VeNKO mice, along with reduced plasma adiponectin concentrations compared with those in their counterparts (Figure 2J,K). We next investigated the role of vascular endothelial NAD^+^ in hemodynamic homeostasis. Male VeNKO mice exhibited elevated SBP and diastolic BP (DBP), cardiac hypertrophy, and reduced arterial remodeling, as evidenced by the increased aortic media thickness to lumen diameter (M/L) ratio (Figure 2L–N and Table S1). Although female VeNKO mice exhibited significant reductions in endothelial NAD^+^ levels (Figure S6A), glucose tolerance and insulin sensitivity were comparable to those in fl/fl controls (Figure S6C–E). In addition, body weight, fat and lean mass (Figure S4A,B), sWAT and vWAT mass (Figure S6F), CD31‐positive areas in sWAT (Figure S6G), and expression levels of angiogenesis genes (Figure S6H) were comparable between female VeNKO and fl/fl controls. Furthermore, we observed no circulatory abnormalities in female VeNKO mice (Figure S6I–K and Table S1).

**FIGURE 3:**
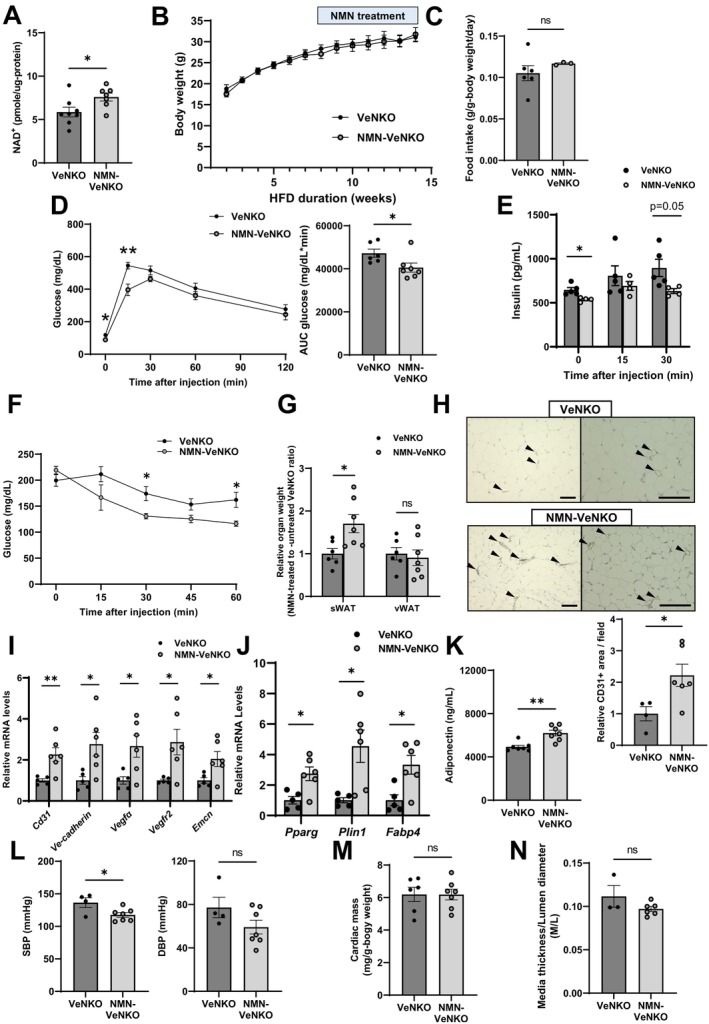
Nicotinamide mononucleotide (NMN) administration improves cardiometabolic functions by increasing angiogenesis. NMN (500 mg/kg body weight/day) was administered via drinking water for up to 9 weeks, starting at week 8 of high‐fat diet feeding in male VeNKO mice. (A) NAD^+^ concentrations in CD31‐positive ECs isolated from the lungs of untreated (black bar) and NMN‐treated (gray bar) VeNKO mice (*n* = 7–8 per group). (B) Body weights of untreated and NMN‐treated VeNKO mice were monitored following initiation of the high‐fat diet (*n* = 6–7 per group). A significant time effect (*p* < 0.05) was observed without any group × time interaction (ANOVA). (C) Daily food intake in untreated and NMN‐treated VeNKO mice after 11–15 weeks of NMN treatment (*n* = 3–6 per group). Changes in blood glucose levels in (D) IPGTTs (*n* = 6–7 per group) and (F) ITTs (*n* = 6 per group). The AUC for glucose during the IPGTT is displayed next to the glucose curves. (E) Plasma insulin concentrations during the IPGTT following overnight fasting (*n* = 4–5 per group). (G) Fat depot masses. Relative fat masses were normalized to the average fat mass in the untreated VeNKO group, which was set as 1 (*n* = 6–7 per group). (H) Representative micrographs of immunohistochemical staining for CD31 in sWAT. Left: Low magnification; Right: High magnification. Arrowheads denote CD31‐positive cells (brown, EC marker). Scale bar, 100 μm. Quantification of CD31‐positive areas per field per mouse (*n* = 4–6 per group). (I) mRNA expression levels of genes involved in angiogenesis in sWAT (*n* = 5–6 per group). (J) mRNA expression of genes involved in adipocyte differentiation in sWAT (*n* = 5–6 per group) and (K) plasma adiponectin levels (*n* = 7 per group). (L) SBP and DBP (mmHg) (*n* = 4–7 per group). (M) Cardiac mass (*n* = 6–7 per group), and (N) ratios of aortic media thickness to lumen diameter (*n* = 3–6 per group). Data were analyzed using Student's unpaired *t*‐test. All values are presented as the mean ± SEM. *p < 0.05, **p < 0.01.

Subsequently, we investigated whether the vascular endothelial *Nampt* deletion‐induced dysfunctions in angiogenesis, adipogenesis, glucose metabolism, and hemodynamic systems were dependent on intracellular NAD^+^ biosynthesis defects. We administered nicotinamide mononucleotide (NMN), a product of NAMPT, via the drinking water of male VeNKO mice fed an HFD for up to 9 weeks (500 mg/kg body weight/day) (Nagahisa et al. 2022; Yamaguchi et al. 2019). NMN administration resulted in a significant increase in vascular endothelial NAD^+^ concentrations, without altering body weight gain or food intake (Figure 3A–C). Notably, NMN administration improved whole‐body glucose intolerance and insulin resistance and decreased insulin concentrations during IPGTTs in VeNKO mice (Figure 3D–F). In addition, NMN administration restored the sWAT mass in VeNKO mice (Figure 3G). Consistently, NMN treatment restored the vascular densities, as quantified by evaluating CD31‐positivity and angiogenesis gene expression (*CD31*, *Ve‐cadherin*, *Vegfα*, *Vegfr2*, and *Emcn*), as well as the expression levels of the adipocyte differentiation markers *Pparg*, *Plin1*, and *Fabp4* in the sWAT of VeNKO mice (Figure 3H–J). NMN administration also restored plasma adiponectin concentration in VeNKO mice (Figure 3K). Although NMN administration significantly lowered SBP, it did not improve DBP, cardiac hypertrophy, or arterial remodeling in VeNKO mice compared with those in age‐matched untreated VeNKO mice (Figure 3L–N). These findings demonstrate that vascular endothelial NAMPT‐mediated NAD^+^ biosynthesis plays important roles in angiogenesis and adipogenesis in sWAT and in cardiometabolic functions, including whole‐body glucose metabolism and BP regulation in HFD‐fed male mice.

### 2.3 Loss of NAMPT Impairs Angiogenesis by Attenuating Endothelial Nitric Oxide Synthase (eNOS) Activity in Vascular Endothelial Cells

**FIGURE 4:**
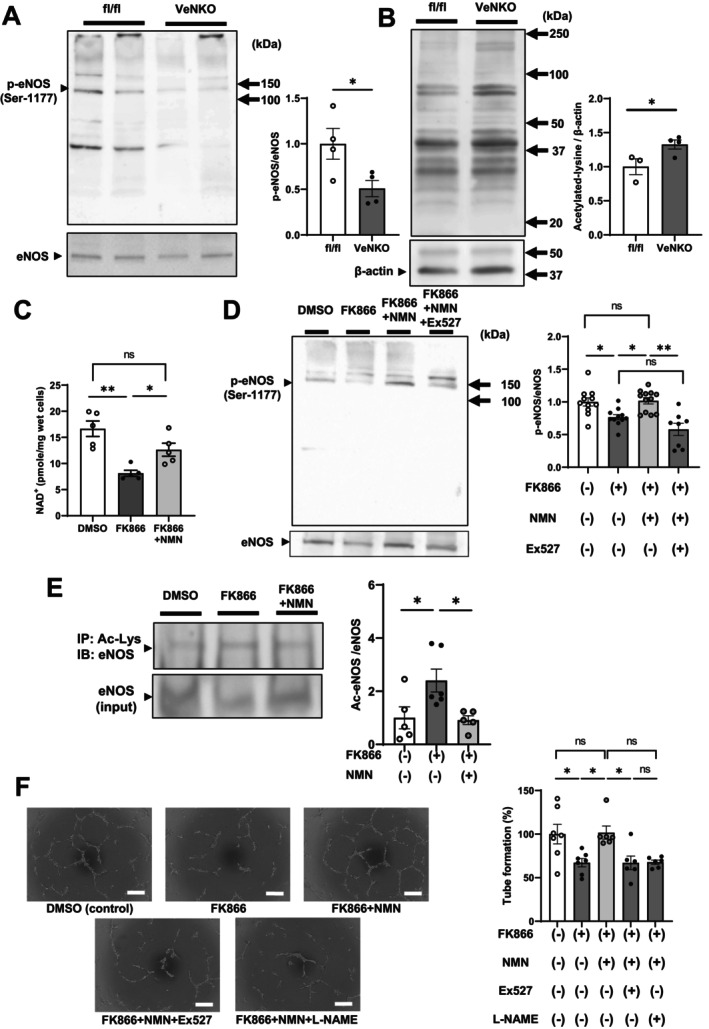
NAMPT loss impairs angiogenesis by attenuating endothelial nitric oxide synthase (eNOS) activity. (A) Western blotting of phosphorylated eNOS at Ser‐1177 (p‐eNOS) and (B) lysine acetylation in the aortas of male VeNKO and fl/fl mice fed an HFD for 15–19 weeks. Band intensities were quantified and normalized to those of native eNOS (*n* = 4 per group) and β‐actin (*n* = 3–4 per group), respectively. (C–F) Human umbilical vein endothelial cells (HUVECs) cultured with or without 0.1% DMSO, 100 nM FK866, a potent NAMPT inhibitor, 10 μM NMN, 10 μM Ex527, a specific inhibitor of the NAD^+^‐dependent deacetylase SIRT1, and 1000 μM NG‐nitro‐L‐arginine methyl ester (L‐NAME), an NOS inhibitor, for 16 h. (C) NAD^+^ concentrations measured in HUVECs with or without FK866 and NMN (*n* = 5 per group). (D) Western blotting of p‐eNOS in HUVECs with or without FK866, NMN, and Ex527 (*n* = 8–12 per group). Band intensities were quantified and normalized to those of native eNOS. (E) Immunoprecipitated acetylated eNOS and input eNOS were evaluated in HUVECs cultured with or without FK866 and NMN. Acetylated eNOS levels were normalized to eNOS protein content (*n* = 5–6 per group). (F) HUVECs cultured in Matrigel to generate capillary‐like structures. Representative micrographs of HUVEC tube assays with or without FK866, NMN, Ex527, and L‐NAME for 16 h (scale bar = 200 μm). The numbers of branches were counted and averaged. Tube formation under each condition was normalized to that of DMSO controls, which was set as 100% (*n* = 6–7 per condition). Data were analyzed using Student's unpaired *t*‐test or ANOVA with Tukey's post hoc test and are presented as the mean ± SEM. *p < 0.05, **p < 0.01.

Phosphorylation of eNOS at Ser‐1177 (p‐eNOS) in the aorta was preserved in HFD‐fed female VeNKO mice (Figure S6B); whereas, we observed a significant reduction in male VeNKO mice compared with fl/fl controls (Figure 4A). In addition, global lysine acetylation was increased in the aortic tissues of male VeNKO mice (Figure 4B). Given that p‐eNOS at Ser‐1177 regulates angiogenesis (Atochin and Huang 2010), we hypothesized that NAD^+^ depletion resulting from NAMPT loss impairs the activity of NAD^+^‐dependent deacetylases, such as sirtuins, thereby reducing eNOS activity in vascular endothelial cells and leading to impaired angiogenesis. To test this hypothesis, we used human umbilical vein endothelial cells (HUVECs), which are a vascular cell line. NAD^+^ concentrations were markedly reduced in HUVECs following treatment with FK866, a potent NAMPT inhibitor, compared with those in untreated control cells; however, NMN treatment partially reversed this effect (Figure 4C). Consistent with the changes in NAD^+^ levels, p‐eNOS at Ser‐1177 levels were significantly reduced in HUVECs following FK866 treatment; NMN treatment normalized these levels. However, Ex527, a specific pharmacological inhibitor of the NAD^+^‐dependent deacetylase SIRT1, abolished the effects of NMN on p‐eNOS at Ser‐1177 levels in FK866‐treated HUVECs (Figure 4D), indicating SIRT1 could be a key mediator of NAMPT‐mediated NAD^+^ biosynthesis in regulating eNOS phosphorylation. In addition, the lysine acetylation levels of eNOS were significantly increased in HUVECs following FK866 treatment; NMN treatment normalized these levels (Figure 4E), indicating that eNOS hyperacetylation could also be involved in the observed decrease in eNOS phosphorylation levels.

To further evaluate the relationships between eNOS activity, the NAMPT–NAD^+^–SIRT1 pathway, and angiogenesis, we performed tube formation assays using Matrigel, which is widely used to model angiogenesis. Consistent with our in vivo observations, pharmacological inhibition of NAMPT decreased tube formation, which was fully rescued by NMN administration. However, the administration of Ex527 and N^G^‐nitro‐L‐arginine methyl ester (L‐NAME), a nitric oxide synthase inhibitor, abrogated the effects of NMN on tube formation in FK866‐treated HUVECs (Figure 4F). We also evaluated whether NMN alone could enhance angiogenesis under physiological conditions. Treatment of HUVECs with NMN alone did not alter NAD^+^ levels, phosphorylation of eNOS at Ser‐1177, or tube formation compared with those in untreated controls (Figure S7A–C), indicating that NMN does not induce the supraphysiological activation of the NAD^+^–SIRT1–eNOS pathway when NAD^+^ homeostasis is intact. Taken together, these findings suggest that eNOS activity acts as a key downstream mediator of NAMPT‐mediated NAD^+^ biosynthesis and the SIRT1 pathway, regulating angiogenesis.

### 2.4 NMN Improves Angiogenesis and Aging‐Associated Complications

**FIGURE 5:**
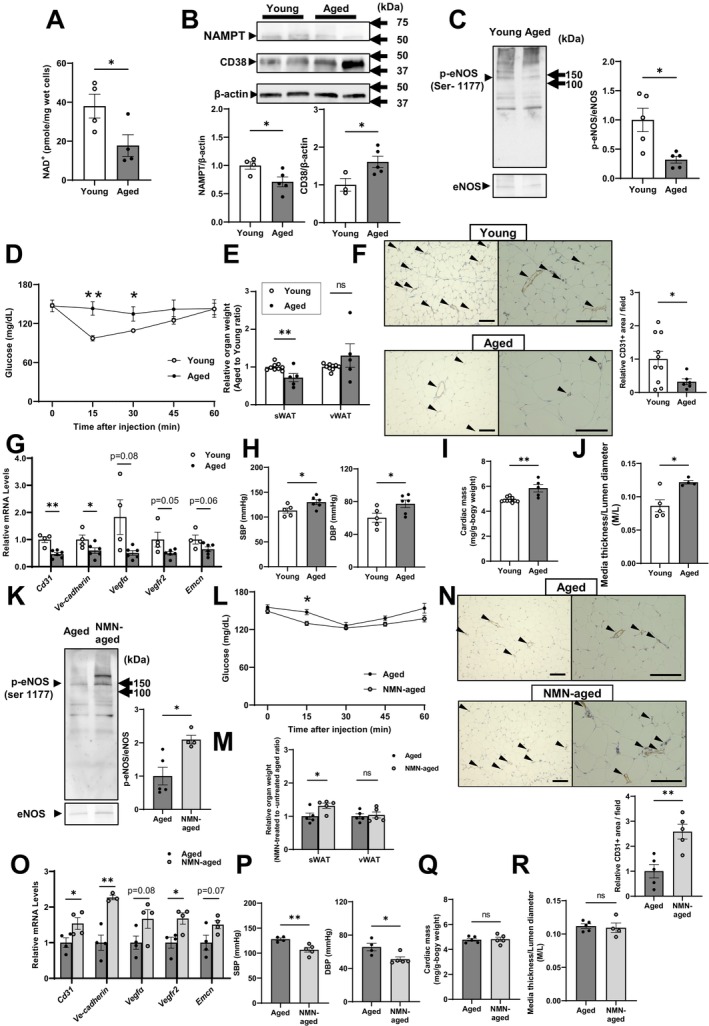
NMN administration ameliorates aging‐associated cardiometabolic complications by increasing angiogenesis. (A–J) C57BL/6 male mice were maintained on a regular chow diet (RCD). Young and aged mice were 2–3 months old (white bar) and 1.5–2 years old (black bar), respectively. (A) NAD^+^ levels in CD31‐positive vascular ECs isolated from the lungs (*n* = 4 per group). (B) Western blotting of NAMPT (*n* = 4–5 per group) and CD38 (*n* = 3–5 per group) in CD31‐positive vascular ECs isolated from the lungs. Band intensities were quantified and normalized to those of β‐actin. (C) Western blotting of aortic phosphorylated endothelial nitric oxide synthase at Ser‐1177 (p‐eNOS) in young and aged male mice. Band intensities were quantified and normalized to those of native eNOS (*n* = 5 per group). (D) Blood glucose concentrations in ITTs (*n* = 5–7 per group). (E) Fat depot masses. Relative fat masses were normalized to the average fat mass in young mice, which was set as 1 (*n* = 5–9 per group). (F) Representative micrographs of immunohistochemical staining for CD31 in sWAT. Left: Low magnification; Right: High magnification. Arrowheads denote CD31‐positive cells (brown, EC marker). Scale bar, 100 μm. Quantification of CD31‐positive vessel densities as CD31‐positive area/field (*n* = 6–10 per group). (G) mRNA expression levels of genes involved in angiogenesis in sWAT (*n* = 4–6 per group). (H) SBP and DBP (mmHg) (*n* = 5–6 per group). (I) Cardiac masses (*n* = 5–10 per group). (J) Ratios of aortic media thickness to lumen diameter (*n* = 4–5 per group). (K–R) The key NAD^+^ intermediate NMN was added (500 mg/kg body weight/day, up to 11 weeks) to the drinking water of aged mice (1.5 years old) fed an RCD. (K) Western blotting of aortic p‐eNOS in untreated (black bar) and NMN‐treated (gray bar) aged male mice. Band intensities were quantified and normalized to those of native eNOS (*n* = 4–5 per group). (L) Blood glucose concentrations in ITTs (*n* = 4 per group). (M) Fat depot masses. Relative fat masses were normalized to the average fat mass in the untreated‐aged group, which was set as 1 (*n* = 5 per group). (N) Representative micrographs of immunohistochemical staining for CD31 in sWAT. Left: Low magnification; right: High magnification. Arrowheads denote CD31‐positive cells (brown, EC marker). Scale bar, 100 μm. Quantification of CD31‐positive areas per field per mouse (*n* = 5 per group). (O) mRNA expression levels of genes involved in angiogenesis in sWAT (*n* = 3–4 per group). (P) SBP and DBP (mmHg, *n* = 4–5 per group), (Q) cardiac mass (*n* = 5 per group), and (R) ratios of aortic media thickness to lumen diameter (*n* = 4–5 per group). Data were analyzed using Student's unpaired *t*‐test. All values are presented as the mean ± SEM. *p < 0.05, **p < 0.01.

Finally, we explored whether vascular endothelial NAD^+^ metabolism is involved in the aging‐associated dysregulation of metabolic and hemodynamic homeostasis. To this end, we examined male 1.5–2‐year‐old mice fed an RCD. NAD^+^ levels, *Nampt* gene, and NAMPT protein expression in CD31‐positive vascular endothelial cells were reduced by 53%, 17%, and 29%, respectively, in the RCD‐fed aged mice compared with those in younger 2–3‐month‐old controls (Figure 5A,B and Figure S8). In addition, the protein expression of NADase CD38, which plays a key role in age‐related NAD^+^ decline (Camacho‐Pereira et al. 2016), was increased by 60% in CD31‐positive vascular endothelial cells from aged mice (Figure 5B). These findings suggest that endothelial NAD^+^ level decline in aged mice reflects both reduced NAMPT expression and increased CD38 expression. These alterations were accompanied by decreased aortic levels of p‐eNOS at Ser‐1177 (Figure 5C). Moreover, in ITTs, insulin injection failed to reduce blood glucose concentrations in aged mice (Figure 5D). Capillary density and the expression of angiogenesis genes were significantly reduced in the sWAT of aged mice compared with those in their younger counterparts (Figure 5F,G). These changes were accompanied by decreased sWAT mass (Figure 5E). In addition, 1.5–2‐year‐old mice exhibited elevated BP, cardiac hypertrophy, and compromised arterial remodeling, as manifested by an increased aortic M/L ratio (Figure 5H–J and Table S2).

We also examined 1.5‐year‐old female C57BL/6J mice fed an RCD to assess whether aging elicits similar alterations in females. NAD^+^ content and NAMPT protein expression in CD31‐positive vascular endothelial cells were moderately reduced by 18% and 25%, respectively, in aged females compared with those in young controls (Figure S9A,B). However, phosphorylation of eNOS at Ser‐1177 remained unchanged with aging in female mice (Figure S9C). Moreover, aged females did not develop insulin resistance as assessed by ITTs, and sWAT and vWAT masses were moderately increased (Figure S9D,E). In addition, the capillary density in sWAT was preserved, as assessed by measuring the CD31‐positive area and angiogenesis gene expression (Figure S9F,G). However, SBP and DBP tended to increase (Figure S9H). No cardiac hypertrophy was observed, and the aortic M/L ratio showed only a modest upward trend (Figure S9I,J and Table S2). These results suggest that although NAMPT expression and NAD^+^ levels decline with aging in female mice, eNOS signaling is preserved, thereby maintaining cardiometabolic homeostasis, in contrast to their male counterparts.

To investigate whether aging‐induced angiogenic, metabolic, and circulatory abnormalities depend on intracellular NAD^+^ metabolism in the vascular endothelium, we added NMN to the drinking water of male 1.5‐year‐old mice for up to 11 weeks. NMN administration (500 mg/kg/day) restored the expression levels of p‐eNOS at Ser‐1177 in the aorta of mice (Figure 5K). In ITTs, the glucose levels of NMN‐treated aged mice were improved (Figure 5L) without changes in their body weight (Figure S10). Compared with untreated aged control mice, NMN‐treated aged mice exhibited substantially larger CD31‐positive areas, higher angiogenesis gene expression (Figure 5N,O), and restored sWAT mass (Figure 5M). NMN administration also decreased both SBP and DBP (Figure 5P) but did not alter cardiac mass and aortic M/L ratio, indicating that these changes were secondary to chronic high BP (Figure 5Q,R). Thus, these findings demonstrate that vascular endothelial NAD^+^ metabolism is disrupted with aging, causing, in males, aging‐associated angiogenic and adipogenic dysfunction in sWAT, as well as abnormalities in glucose metabolism and BP regulation.

## 3 Discussion

In this study, we identified vascular endothelial NAD^+^ as a key regulator of angiogenesis, which, in turn, governs whole‐body glucose metabolism and BP regulation. We investigated this role using VeNKO and naturally aged mice. We found that *Nampt* deletion in vascular endothelial cells impaired angiogenesis, leading to defective adipogenesis and subcutaneous fat loss, contributing to abnormalities in whole‐body glucose metabolism and BP regulation. Mechanistically, NAMPT deletion attenuated eNOS activity and angiogenesis, likely through the disruption of the NAD^+^–SIRT1–eNOS axis. Furthermore, the naturally aged mice exhibited a substantial reduction in vascular endothelial NAD^+^ levels, accompanied by impaired eNOS signaling and angiogenesis. Aging‐associated disorders, including sWAT loss, whole‐body insulin resistance, and elevated BP, supplemented these changes. Finally, administration of NMN, a key NAD^+^ intermediate, reinstated eNOS activity and angiogenesis, ameliorated these aging‐associated metabolic disorders, and lowered high BP. Collectively, these findings highlight the critical role of the vascular endothelial NAMPT–NAD^+^–SIRT1–eNOS signaling axis in angiogenesis, which, in turn, regulates adipogenesis and whole‐body glucose metabolism in conjunction with BP homeostasis; our study also highlights the therapeutic potential of NMN for aging‐associated cardiometabolic complications.

Our results underscore the importance of vascular endothelial NAD^+^ biosynthesis in angiogenesis. Previous in vitro studies demonstrated that vascular endothelial NAMPT‐mediated NAD^+^ biosynthesis is compromised during replicative aging, affecting angiogenesis (Borradaile and Pickering 2009). The reduction in endothelial NAD^+^ levels suppresses ATP production, restricting the energy supply needed for endothelial cell proliferation, whereas NMN replenishes NAD^+^ and enhances angiogenic potential in aged mice (Kiesworo et al. 2024), indicating that NAD^+^ biology is critically involved in aging‐associated angiogenesis decline. Although still undetermined, the precise molecular mechanism of NAD^+^‐mediated angiogenesis likely involves eNOS activity. eNOS phosphorylation plays a crucial role in regulating eNOS activity, endothelial functions, and angiogenesis (Atochin and Huang 2010). Our mechanistic studies revealed that NAD^+^ depletion increases eNOS acetylation in vitro, an effect reversed by NMN supplementation, confirming that intracellular NAD^+^ levels regulate eNOS acetylation via the NAMPT–NAD^+^–sirtuin pathway. We further showed that NAMPT–NAD^+^–SIRT1 regulates eNOS phosphorylation at Ser‐1177 and regulates angiogenesis as assessed by evaluating tube formation in vitro. Consistently, NAD^+^ depletion was associated with increased global lysine acetylation and reduced eNOS phosphorylation at Ser‐1177 in the aorta of VeNKO mice. These findings collectively indicate that NAMPT‐mediated NAD^+^ biosynthesis and SIRT1 regulate eNOS activity by modulating its phosphorylation and acetylation status, thereby controlling endothelial angiogenic capacity. This is consistent with previous studies demonstrating that SIRT1 directly deacetylates eNOS to enhance its enzymatic activity (Mattagajasingh et al. 2007) and indirectly promotes eNOS phosphorylation at Ser‐1177 via deacetylation of liver kinase B1, which activates AMPK (Chen et al. 2010; Lan et al. 2008). Importantly, these mechanisms are interdependent: AMPK‐mediated phosphorylation of eNOS at Ser‐1177 facilitates SIRT1 binding and subsequent deacetylation (Chen et al. 2010). Together, these processes define a tightly integrated regulatory network through which the NAD^+^‐dependent deacetylase SIRT1 maintains eNOS activity by coordinating its acetylation and phosphorylation status.

We assessed the angiogenic capacity of endothelial cells with a particular focus on microvascular angiogenesis in adipose tissue. Angiogenesis is closely associated with adipogenesis, with vascular endothelial cells in adipose tissue participating in adipocyte differentiation and adipose function (Christiaens and Lijnen 2009; Sung et al. 2013). Aging diminishes angiogenic capacity in WAT (Atochin and Huang 2010). Impaired angiogenesis and adipogenesis contribute to aging‐associated sWAT dysfunction and volume loss, exacerbating glucose metabolism and mortality (Palmer and Jensen 2022; Patel and Abate 2013). For example, *Sirt1* deletion attenuates angiogenesis in adipose tissue, deteriorating adipogenesis (Xu et al. 2012). Adipocyte‐specific depletion of vascular endothelial growth factor, a key angiogenic factor secreted by adipose tissue, compromises adipose tissue angiogenesis and blood perfusion in a paracrine manner, promoting systemic metabolic dysfunction and insulin resistance (Sung et al. 2013). This finding highlights the critical role of adequate vascular support in maintaining adipose tissue function and systemic metabolic homeostasis. In our study, vascular endothelial NAD^+^ deficiency was associated with impaired angiogenesis in sWAT, accompanied by subcutaneous adipogenic dysfunction, fat volume loss, and systemic metabolic dysfunction. Consistent with these findings, VeNKO mice exhibited a significant reduction in phosphorylated Akt at Ser‐473 in sWAT. Although vascular endothelial *Nampt* deletion reduced plasma adiponectin levels, phosphorylated Akt at Ser‐473 and triglyceride levels in the hepatic and skeletal muscles were not altered, indicating a tissue‐specific impairment of insulin sensitivity localized to adipose tissue (Samuel and Shulman 2016). However, because Akt phosphorylation was evaluated under noninsulin‐stimulated conditions, the potential contribution of skeletal muscles or the liver to whole‐body glucose metabolism abnormalities cannot be entirely disregarded. Moreover, endothelial insulin resistance may have contributed to the whole‐body glucose metabolism abnormalities observed in VeNKO mice, as endothelial eNOS phosphorylation at Ser‐1177 is regulated by Akt signaling (Dimmeler et al. 1999) and *Sirt3* deficiency in vascular endothelial cells induces endothelial insulin resistance (Yang et al. 2016).

Despite reduced NAD^+^ levels in vessels supplying blood to both sWAT and vWAT in VeNKO mice, angiogenesis impairment was observed only in sWAT. A possible explanation for this depot‐specific effect is the inherently greater angiogenic capacity of vascular endothelial cells in sWAT compared with vWAT (Gealekman et al. 2011; Patel and Abate 2013), rendering subcutaneous vessels more vulnerable to NAD^+^ depletion. Additionally, the distinct effects of NMN treatment on sWAT and vWAT depots support the notion that vascular NAD^+^ deficiency influences angiogenesis in a depot‐specific manner (Imi et al. 2023). Thus, our data suggest that the NAMPT–NAD^+^–SIRT1–eNOS signaling axis plays a key role in maintaining subcutaneous fat volume and whole‐body glucose metabolism by regulating angiogenesis and adipogenesis in sWAT.

Besides its role in metabolic regulation, vascular NAD^+^ metabolism contributes to hemodynamics. eNOS knockout mice reportedly exhibit vascular rarefaction, likely responsible for high BP (Kubis et al. 2002). Thus, the BP elevation in VeNKO mice observed in our study could be attributed to impaired angiogenesis induced by NAMPT deletion and eNOS inactivation. In VeNKO mice, high BP was accompanied by cardiac hypertrophy and reduced arterial remodeling. However, NMN administration lowered high BP but did not reverse cardiac or arterial derangements; these findings are consistent with those from previous studies reporting cardiac hypertrophy (Lapu‐Bula and Ofili 2007) and reduced arterial remodeling (de Ciuceis et al. 2005) as secondary complications of vascular endothelial dysfunction. Additionally, sirtuins may contribute to the hemodynamic dysregulation observed in VeNKO mice, as endothelial cell‐specific *Sirt6* knockout mice exhibited elevated BP following Ang‐II administration and increased left ventricular hypertrophy and arterial thickening under desoxycorticosterone acetate/salt treatment (Guo et al. 2019). Although endothelium‐dependent vasorelaxation was not directly assessed in our study, eNOS knockout mice displayed hypertension due to the lack of vascular relaxation (Atochin and Huang 2010), whereas NMN‐treated aged mice exhibited improved aortic endothelial NO‐mediated vasodilation (Tarantini et al. 2019). Therefore, endothelium‐dependent vasorelaxation dysfunction may contribute to elevated BP in VeNKO and aged mice. Thus, the vascular NAMPT–NAD^+^–SIRT1–eNOS axis regulates angiogenesis, which, in turn, governs hemodynamics.

Our study demonstrates sex‐specific differences in the consequences of endothelial NAD^+^ depletion. Despite equivalent reductions in endothelial NAD^+^ levels in both sexes, only male VeNKO and aged mice exhibited diminished eNOS phosphorylation at Ser‐1177, impaired angiogenesis, and consequent cardiometabolic dysfunction. In contrast, in female mice, the eNOS phosphorylation levels and normal metabolic and hemodynamic functions were maintained, indicating a female‐specific resilience. Mechanistically, estrogen is likely to provide this protection through complementary pathways. Estrogen preserves eNOS phosphorylation at Ser‐1177 by promoting SIRT1 expression (Tsuchiya et al. 2020) and independently activating the PI3K–Akt pathway (Haynes et al. 2000). Importantly, these mechanisms likely persist, at least partially, even as estrogen levels decline with aging, contributing to the relative protection observed in females. Indeed, aging‐associated endothelial dysfunction and metabolic impairment are more pronounced in males (Cao et al. 2017), whereas residual estrogen signaling may continue to preserve vascular homeostasis in aged females (Miller and Duckles 2008). These insights align with prior reports of greater angiogenic capacity (Rudnicki et al. 2018) and subcutaneous adipose volume maintenance (Blaak 2001) in female mice during metabolic stress, consistent with the estrogen‐induced preferential promotion of angiogenesis and adipogenesis in subcutaneous depots (Dieudonne et al. 2000). Collectively, our findings indicate that estrogen‐dependent preservation of eNOS signaling is a key mechanism underlying sex‐specific resilience to endothelial NAD^+^ depletion, with direct implications for the pathophysiology and treatment of aging‐associated cardiometabolic diseases.

Although the molecular mechanisms underlying aging‐induced impairment of vascular endothelial NAD^+^ metabolism remain elusive, this study provides mechanistic insight into the decline of endothelial NAD^+^ levels in aged male mice. We showed that aging induces NAD^+^ depletion in endothelial cells through two complementary processes: reduced NAMPT‐mediated biosynthesis and increased NAD^+^ catabolism driven by CD38 upregulation. CD38, a key NAD^+^‐consuming enzyme, is abundantly expressed in endothelial cells (Boslett et al. 2017), and its expression is markedly increased with aging across multiple tissues, contributing to systemic NAD^+^ decline (Camacho‐Pereira et al. 2016). This dual impairment of NAD^+^ metabolism may critically contribute to the reduced angiogenic capacity and vascular dysfunction associated with aging.

Our study suggests that targeting vascular NAD^+^ metabolism may provide a novel therapeutic approach for aging‐associated cardiometabolic dysfunction. sWAT volume, which is closely linked to angiogenesis, declines with age (Coín Aragüez et al. 2012; Palmer and Jensen 2022), and this reduction contributes to glucose metabolism abnormalities, cardiovascular disease, and increased mortality (Murphy et al. 2013; Patel and Abate 2013). Maintaining vascular NAD^+^ metabolism may therefore be crucial for preserving subcutaneous fat mass and promoting longevity. Supporting this concept, NMN was shown to restore NO‐dependent endothelial function in aged mice (de Picciotto et al. 2016) and mitigate arterial stiffness in middle‐aged humans (Katayoshi et al. 2023). Moreover, estrogen may play a protective role against cardiometabolic dysfunction driven by vascular NAD^+^ decline, suggesting that postmenopausal women may be more vulnerable to vascular NAD^+^ depletion‐associated cardiometabolic diseases because of lower estrogen levels. Further research is warranted to elucidate the role of vascular NAD^+^ depletion in cardiometabolic dysfunction in aging males and postmenopausal females, with the aim of informing sex‐specific therapeutic strategies targeting NAD^+^ metabolism.

In conclusion, our study identifies vascular NAD^+^ metabolism as a central regulator of angiogenesis and adipogenesis, essential for maintaining cardiometabolic homeostasis during aging. These findings highlight the NAMPT–NAD^+^–SIRT1–eNOS signaling axis as a critical mechanistic link connecting vascular health to metabolic and hemodynamic regulation. Given its potential to boost vascular NAD^+^ metabolism, urgent investigation of the role of NMN is warranted to develop a therapeutic approach for preventing or reversing aging‐associated cardiometabolic dysfunction and reducing the overall mortality risk.

## 4 Materials and Methods

### 4.1 Animal Experiments

VeNKO mice were generated by crossing *Ve‐cadherin*‐Cre transgenic mice (006137; Jackson Laboratory, Bar Harbor, ME, USA) with floxed‐*Nampt* (fl/fl) mice (Nagahisa et al. 2022; Stromsdorfer et al. 2016; Yamaguchi et al. 2019). Mice were housed under specific pathogen‐free conditions and a 12‐h light/dark cycle and provided with RCD (CE‐2; CLEA Japan Inc., Tokyo, Japan) or HFD (45% kcal from fat; D12451, Research Diets Inc., New Brunswick, NJ, USA), with *ad libitum* access to water. C57BL/6J male and female mice were purchased from CLEA Japan (Tokyo, Japan) and maintained on an RCD. Briefly, 2–3‐month‐old and 1.5–2‐year‐old mice were defined as young and aged mice, respectively. Aged mice (2 years old) were provided by the Foundation for Biomedical Research and Innovation at Kobe through the National BioResource Project of the MEXT, Japan. Murine tissue samples were quick‐frozen in liquid nitrogen and stored at −80°C until further use. Plasma adiponectin levels were measured using a commercial ELISA kit (MRP300, R&D Systems Inc., Minneapolis, MN, USA). All animal procedures were approved (A2022‐259) by the Institutional Animal Care and Use Committee of Keio University School of Medicine.

### 4.2 Glucose and Energy Metabolism

For IPGTTs, 50% dextrose solution (2 g/kg body weight) was administered intraperitoneally to mice after ~16 h of fasting. For ITTs, mice were injected intraperitoneally with human insulin (0.75 U/kg body weight) after fasting for 4 h (Yamaguchi et al. 2019). Blood glucose levels were measured at 0, 15, 30, 60, and 120 min after glucose loading and at 0, 15, 30, and 60 min after insulin injection using the OneTouch Ultra View blood glucose meter (Johnson & Johnson KK, Tokyo, Japan). Metabolic phenotypes were assessed in multiple cohorts. Plasma insulin concentrations were determined using commercially available ELISA kits (#M1104; Morinaga Institute of Biological Science, Yokohama, Japan). Food intake was assessed for each mouse under individually housed conditions, including a 1‐day acclimation period followed by measurements taken over four consecutive days. For each day, food intake (g/day) was calculated by subtracting the residual chow weight from the initial amount provided and then divided by the corresponding body weight to obtain the daily intake normalized to body weight (g/g‐body weight/day). The normalized values from the 4‐day measurement period were averaged to derive the mean normalized daily intake for each mouse (g/g‐body weight/day). Metabolic parameters were measured using a biogas analytical mass spectrometer (ARCO‐2000; Arco System, Chiba, Japan). Briefly, mice were individually housed and monitored under a 12‐h light/dark cycle with *ad libitum* access to water and food for 3 days, including 1 day of acclimation. Whole‐body oxygen consumption, CO_2_ production, and the respiratory exchange ratio were measured, and energy expenditure was calculated and expressed per mouse.

### 4.3 NMN Rescue

NMN (#44501900, Oriental Yeast Co., Tokyo, Japan), at a dose of 500 mg/kg body weight/day (Nagahisa et al. 2022; Stromsdorfer et al. 2016; Yamaguchi et al. 2019), was added to the drinking water of male VeNKO mice fed an HFD for up to 9 weeks and that of 1.5‐year‐old C57BL/6J male mice fed an RCD for up to 11 weeks (NMN‐treated group).

### 4.4 Histology

Adipose and aorta tissue samples were fixed in 4% paraformaldehyde phosphate buffer solution, embedded in paraffin, and cut into 5‐μm and 3‐μm‐thick sections, respectively. Hematoxylin and eosin staining or immunostaining using anti‐CD31 antibodies (ab28364; Abcam, Cambridge, UK) was performed at the Institute of Nutrition & Pathology Inc. (Kyoto, Japan). Images were captured using an Axiocam camera or an all‐in‐one fluorescence microscope (BZ‐X800, Keyence, Osaka, Japan), and analyzed using the AxioVision software (Carl Zeiss, Göttingen, Germany). To quantify blood vessels in adipose tissue, at least 10 fields of view were randomly imaged; CD31‐positive areas were quantified using ImageJ (National Institutes of Health, Bethesda, MD, USA).

### 4.5 Real‐Time PCR

Total RNA was isolated using the RNeasy Plus Mini Kit (#74134; Qiagen, Valencia, CA, USA) or TRIzol reagent (#15596018; Invitrogen, Carlsbad, CA, USA), then reverse‐transcribed into cDNA using the PrimeScript RT Master Mix (#RR036B; Takara Bio, Shiga, Japan). Relative gene expression levels were measured using the KAPA SYBR FAST Universal Kit (#KK4602; Sigma‐Aldrich, St. Louis, MO, USA). Expression was quantified by normalizing the cycle threshold values to those of the housekeeping control gene encoding a ribosomal protein (*36b4*) (Nagahisa et al. 2022). The primers used are listed in Table S3.

### 4.6 BP Assessment

BP of unanesthetized mice was measured using a tail‐cuff system (MK‐2000st, Muromachi Kikai, Tokyo, Japan) during the light phase. At least three readings were obtained for each measurement; the final value was calculated as the mean of all data. SBP and mean blood pressure (MBP) were measured, and DBP was calculated using the formula: (3 × MBP − SBP)/2. Mice were acclimated to the apparatus prior to analysis. BP was assessed in multiple cohorts.

### 4.7 Endothelial Cells

Endothelial cells were isolated from the lungs, sWAT, and vWAT, as previously described (Kanda et al. 2008). Briefly, tissues were washed in Dulbecco's Modified Eagle Medium (DMEM) (#11995‐065; Gibco, Carlsbad, CA, USA) containing 20% fetal bovine serum, minced finely with scissors, and digested in collagenase (Sigma‐Aldrich) at 37°C for 30 min. The resulting suspension was dissociated, filtered through a 70‐μm strainer, and pelleted at 300 *× g* for 5 min. The pellet was resuspended and incubated with CD31‐coated magnetic beads (#11035; Thermo Fisher Scientific, Waltham, MA, USA) at 4°C for 20 min with gentle rotation. A magnetic separator was used to isolate bead‐bound cells.

### 4.8 Bone Marrow Cell Isolation

Hematopoietic lineage cells included in total bone marrow cell isolates were obtained from mouse femurs and tibias. Briefly, femurs and tibias were excised, cleaned of connective tissue, and placed into a 0.5‐mL microcentrifuge tube with an 18G needle puncture at the bottom, nested within a 1.5‐mL tube. The assembly was centrifuged at 10,000 × *g* for 15 s to isolate bone marrow cells into the outer tube. The collected cells were resuspended in ACK lysis buffer (#A1049201; Gibco, Carlsbad, CA, USA), mixed gently, washed with phosphate‐buffered saline (PBS), and centrifuged at 300 × *g* for 5 min to obtain bone marrow cells for analysis.

### 4.9 Cell Culture

Briefly, HUVECs (C2517A, Lonza, Switzerland) were cultured in EGM‐2 medium (#CC‐3156 and #CC‐4176; Lonza, Allendale, NJ, USA) at 37°C and 5% CO_2_. To assess the expression of eNOS and p‐eNOS, HUVECs were incubated in serum‐free medium with or without 0.1% dimethyl sulfoxide (DMSO), 100 nM FK866 (Cayman Chemical, Ann Arbor, MI, USA), 10 μM Ex527 (#E7034; Sigma‐Aldrich), and 10 μM NMN (Sigma‐Aldrich) for 16 h.

### 4.10 Tube Formation Assay

HUVECs were cultured on Geltrex LDEV‐free reduced growth factor basement membrane matrix‐coated (#A1413201; Thermo Fisher Scientific) 96‐well plates at a density of 120,000 cells/cm^2^ in serum‐free EGM‐2 medium with or without 0.1% DMSO, 100 nM FK866, 10 μM NMN, 10 μM Ex527 (#E7034; Sigma‐Aldrich), and 1000 μM N^G^‐nitro‐L‐arginine methyl ester (L‐NAME; #HY‐18729A, MedChemExpress, Monmouth Junction, NJ, USA) for 16 h at 37°C and 5% CO_2_. Three images were captured per well, and the mean numbers of branches were quantified. Tube formation in the control sample was considered 100%. Results from experimental groups were expressed as a percentage relative to this reference.

### 4.11 NAD ^+^ Assays

NAD^+^ was extracted from frozen tissue samples and HUVECs using ice‐cold perchloric acid and neutralized with potassium carbonate. Concentrations were determined using an HPLC system (Prominence; Shimadzu Scientific Instruments, Kyoto, Japan) fitted with a Supelco LC‐18‐T column (#58970‐U; Sigma‐Aldrich), as previously described (Nagahisa et al. 2022; Yamaguchi et al. 2019; Yamaguchi et al. 2024). NAD^+^ concentrations were normalized to tissue weight, wet‐cell weight, or total protein content. Wet‐cell weight was calculated as the difference between the weight of the Eppendorf tube before and after adding the cell pellet (Yamaguchi et al. 2024). Total protein content was measured by dissolving the pellet obtained after acid treatment in radioimmunoprecipitation assay buffer (#182–02451; FUJIFILM Wako Pure Chemical, Osaka, Japan) using the BCA Protein Assay Kit (#23225; Thermo Fisher Scientific).

### 4.12 Triglyceride Quantification

Approximately 100 mg of tissue was homogenized in 1 mL of chloroform: methanol (2:1, v/v), followed by the addition of 200 μL of 50 mM sodium chloride to facilitate phase separation. The mixture was centrifuged at 100 × *g* for 5 min, and the lower organic phase was collected and dried. The resulting lipid extract was reconstituted in 10% Triton X‐100 for samples derived from liver or skeletal muscle, or in isopropanol for those derived from sWAT. Triglyceride levels were determined using a commercially available assay kit (#291‐94,501, FUJIFILM Wako Pure Chemical Corporation, Osaka, Japan), according to the manufacturer's protocol.

### 4.13 Western Blotting

Total protein was extracted from frozen tissues homogenized in radioimmunoprecipitation assay buffer containing protease and phosphatase inhibitors (#5872; Cell Signaling Technology, Danvers, MA, USA). Extracted protein samples were separated using sodium dodecyl sulfate–polyacrylamide gel electrophoresis and transferred to polyvinylidene difluoride membranes (#88518; Thermo Fisher Scientific). Blocking and antibody dilution solutions consisted of skim milk or commercially available buffer (#NKB101/NYPBR; TOYOBO Co. Ltd., Osaka, Japan). Membranes were probed using the following primary antibodies: rabbit monoclonal anti‐NAMPT (#86634; Cell Signaling Technology; dilution: 1:500), rabbit monoclonal anti‐eNOS (#32027; Cell Signaling Technology; dilution: 1:250), rabbit polyclonal anti‐p‐eNOS (Ser1177) (#9571; Cell Signaling Technology; dilution: 1:250), rabbit monoclonal anti‐Akt (pan) (C67E7) (#4691; Cell Signaling Technology; dilution: 1:1000), rabbit monoclonal anti‐phospho‐Akt (Ser473) (D9E) XP (#4060; Cell Signaling Technology; dilution: 1:2000), rabbit polyclonal anti‐acetylated lysine antibody (#9441; Cell Signaling Technology; dilution: 1:1000), rabbit monoclonal anti‐CD38 (E9F5A) XP (#68336; Cell Signaling Technology; dilution: 1:1000), and mouse monoclonal anti‐β‐actin (#A5316; Sigma‐Aldrich; dilution: 1:5000). Subsequently, all blots were incubated with horseradish peroxidase (HRP)‐conjugated anti‐rabbit (#111‐035‐003; Jackson ImmunoResearch Laboratories, West Grove, PA, USA; dilution: 1:2000) or HRP–conjugated anti‐mouse (#115‐035‐003; Jackson ImmunoResearch Laboratories; dilution: 1:2000) secondary IgG. Reactivity was visualized using the ECL Prime Western Blotting Detection Reagent (#RPN2232; GE Healthcare, Pittsburgh, PA, USA). Blots used for detecting NAMPT, CD38, acetylated lysine, p‐eNOS, or p‐Akt were stripped and reprobed with anti‐β‐actin, ‐eNOS, or ‐Akt antibodies for normalization, respectively. Densitometry was performed using the ImageJ software (National Institutes of Health).

### 4.14 Immunoprecipitation

Protein samples for detecting acetylated eNOS were prepared as previously described (Stromsdorfer et al. 2016). HUVEC samples were collected after incubation with 0.1% DMSO, 100 nM FK866, and 10 μM NMN for 16 h at 37°C and 5% CO_2_, rinsed in ice‐cold PBS containing 1 μM trichostatin A (#9950; Cell Signaling Technology) and 10 mM nicotinamide (#72340; Sigma‐Aldrich), and immediately frozen. Frozen cell samples were homogenized in acKIP buffer containing phosphatase and protease inhibitor cocktail (04693116001; Roche Diagnostics, Berlin, Germany), 1 μM trichostatin A, and 10 mM nicotinamide. Protein samples (1.0 mg) were subjected to immunoprecipitation using anti‐acetylated lysine agarose beads (#ICP0388; Immunochem Pharmaceuticals, Burnaby, BC, Canada). Immunoprecipitated acetylated eNOS and input eNOS were detected using western blotting with rabbit monoclonal anti‐eNOS (#32027; Cell Signaling Technology; dilution: 1:200) and HRP‐conjugated anti‐rabbit (#111–035‐003; Jackson ImmunoResearch Laboratories; dilution: 1:2000) as described above.

### 4.15 Quantification and Statistical Analysis

Data are presented as the mean ± standard error of the mean (SEM). Statistical analyses were conducted using GraphPad Prism (version 9.5; GraphPad Software, San Diego, CA, USA). Differences between groups were assessed using Student's unpaired *t‐*test. Comparisons among multiple (> 2) groups were conducted using one‐way analysis of variance (ANOVA) with Tukey's post hoc test. Differences in continuous metabolic parameters (e.g., body weight) were assessed using repeated measures ANOVA. Results with *p* < 0.05 were considered statistically significant.

## Author Contributions

S.Y. conceived and designed the study. S.K., S.Y., K.N., T.N., and Y.W. performed the experiments. S.K. and S.Y. wrote the manuscript. D.K., K.K., R.M., R.N., K.H., T.K., J.I., K.M., and T.M. assisted with the editing of the manuscript. Y.K. and K.K. provided scientific suggestions and assisted with the editing of the manuscript. J.Y., H.I., and K.H. provided scientific suggestions, supervision, and detailed comments on the manuscript. All authors reviewed and edited the manuscript.

## Ethics Statement

All animal procedures were approved (A2022‐259) by the Institutional Animal Care and Use Committee of Keio University School of Medicine.

## Conflicts of Interest

The authors declare no conflicts of interest.

## Supporting information


**Figure S1:** **Ve‐cadherin‐Cre transgenic mice exhibit Nampt expression levels comparable to those observed in fl/fl mice.** Quantification of *Nampt* expression in CD31‐positive endothelial cells isolated from the visceral white adipose (vWAT) tissue of 2‐month‐old *Ve‐cadherin*‐Cre transgenic (Cre) and control (fl/fl) male mice (*n* = 4 per group). Data were analyzed using Student's unpaired *t*‐test. All data are presented as the mean ± standard error of the mean (SEM).

**Figure S2:** **VeNKO** **mice maintain *Nampt* expression in hematopoietic lineage cells similar to fl/fl mice.** *Nampt* mRNA levels were quantitatively assessed in hematopoietic lineage cells included in total bone marrow cell isolates obtained from 2‐month‐old fl/fl and VeNKO male mice (*n* = 3–6 per group). Data were analyzed using Student's unpaired *t*‐test. All data are presented as the mean ± SEM.

**Figure S3:** **Vascular endothelial cell‐specific *Nampt* deletion has minimal effect on energy and glucose metabolism and cardiovascular homeostasis in mice fed a regular chow diet (RCD).** Characterization of fl/fl and vascular endothelial cell‐specific *Nampt* knockout (VeNKO) mice fed an RCD. (A) Body weights of fl/fl and VeNKO mice. Body weights of male (*n* = 6–10 per group) and female (*n* = 12–13 per group) mice were monitored soon after weaning. A significant time effect (*p* < 0.05) was observed without any group × time interaction (ANOVA). (B) Body composition and organ weights: liver, kidneys, heart, subcutaneous white adipose tissue (sWAT), vWAT, and brown adipose tissue (BAT), were measured in 5–6‐month‐old female mice (*n* = 5 per group). (C) Daily food intake in 2–3‐month‐old female mice (*n* = 5 per group). (D) Respiratory quotient and energy expenditure in 2–3‐month‐old female mice (*n* = 4–5 per group). Blood glucose concentrations measured using (E) glucose (*n* = 5–16 per group) and (F) insulin (*n* = 4–8 per group) tolerance tests in 2–5‐month‐old male and female mice. The area under the curve (AUC) for glucose during the glucose tolerance test is displayed alongside the glucose curves for both males and females. (G) Systolic blood pressure (SBP) (mmHg) in 3–5‐month‐old male and female mice (*n* = 4–6 per group). (H) Cardiac masses in 5–6‐month‐old female mice (*n* = 5 per group). Data were analyzed using Student's unpaired *t*‐test. All values are presented as the mean ± SEM.

**Figure S4:** ***Nampt* deficiency in vascular endothelial cells has minimal effect on body weight and composition in mice fed a high‐fat diet (HFD).** Both fl/fl and VeNKO mice were fed an HFD. (A) Body weights (*n* = 7–13 per group) and (B) body composition (*n* = 7–11 per group) were measured in 4–5‐month‐old VeNKO and fl/fl male and female mice. Data were analyzed using Student's unpaired *t*‐test. All values are presented as the mean ± SEM.

**Figure S5:** **Vascular endothelial NAMPT deficiency affects insulin signaling in sWAT, without impairing liver or skeletal muscle metabolism in HFD‐fed VeNKO mice.** Western blot analysis of levels of phosphorylated Akt at Ser473 (p‐Akt) in the (A) liver (*n* = 4–5 per group), (C) skeletal muscle (*n* = 4–5 per group), and (E) sWAT (*n* = 4–5 per group) after 15–19 weeks of HFD feeding. Band intensities were quantified and normalized to total Akt levels. Triglyceride levels in the (B) liver (*n* = 5 per group), (D) skeletal muscle (*n* = 6–7 per group), and (F) sWAT (*n* = 4 per group) were measured after 14–17 weeks of HFD feeding. Data were analyzed using unpaired Student's *t*‐test. Values are presented as the mean ± SEM. *p < 0.05.

**Figure S6:** **Vascular endothelial cell‐specific** ***Nampt* deletion in female mice fed a HFD has minimal effect on glucose metabolism and cardiovascular homeostasis.** (A) NAD^+^ levels in endothelial cells isolated from the lungs of mice after 16–19 weeks of HFD (*n* = 5–8 per group). (B) Western blotting of levels of phosphorylated endothelial nitric oxide synthase (eNOS) at Ser‐1177 (p‐eNOS) in the aortas of female VeNKO and fl/fl mice fed HFD for 16–19 weeks. Band intensities were quantified and normalized to those of native eNOS (*n* = 3–5 per group). (C) Glucose (*n* = 7–11 per group) and (E) insulin (*n* = 6–11 per group) tolerance test results performed after 10–13 weeks of HFD. The AUC for glucose during the glucose tolerance test is displayed next to the glucose curves. (D) Plasma insulin concentrations during the glucose tolerance tests following overnight fasting (*n* = 4–5 per group). (F) Evaluation of fat depot masses, including sWAT and vWAT, after 16–19 weeks of HFD feeding. Relative fat mass was normalized to that of the fl/fl group, which was set as 1 (*n* = 7–9 per group). (G) Representative micrographs of immunohistochemical staining for CD31 in sWAT after 16–19 weeks of HFD feeding (*n* = 5 per group). Left: low magnification; right: high magnification. Arrowheads denote CD31‐positive cells (brown, endothelial cell marker). Scale bar, 100 μm. Quantification of CD31‐positive areas per field per mouse (*n* = 5 per group). (H) mRNA expression levels of genes involved in angiogenesis in sWAT after 15–19 weeks of HFD feeding (*n* = 5–7 per group). (I) SBP and diastolic blood pressure (DBP) (mmHg) measured after 15–16 weeks of HFD (*n* = 7–8 per group). (J) Cardiac masses after 16–19 weeks of HFD (*n* = 7–11 per group). (K) Aortic media thickness/lumen diameter (*n* = 5 per group). Data were analyzed using Student's unpaired *t*‐test. All values are presented as the mean ± SEM. **p < 0.01.

**Figure S7:** **NMN supplementation alone does not enhance eNOS or angiogenic activity in endothelial cells.** (A–C) Human umbilical vein endothelial cells (HUVECs) were cultured for 16 h in the presence of either water (vehicle control, white bars) or 10 μM NMN (blue bars). (A) NAD^+^ concentrations measured following treatment (*n* = 5–6 per group). (B) Western blot analysis of levels of p‐eNOS in HUVECs (*n* = 4 per group). Band intensities of p‐eNOS were normalized to total eNOS levels. (C) HUVECs were cultured in Matrigel to generate capillary‐like structures. Representative micrographs of HUVEC tube assays in the presence of either water or NMN for 16 h (scale bar = 200 μm). The branches were counted and averaged. Tube formation under NMN‐treatment was normalized to that of the control group, which was set as 100% (*n* = 4 per condition). Data were analyzed using Student's unpaired *t*‐test. All values are presented as the mean ± SEM.

**Figure S8:** **Aging reduces *Nampt* expression in endothelial cells.** CD31‐positive endothelial cells were isolated from white adipose tissue of young (2–3 months; white bars) and aged (18–24 months; black bars) C57BL/6 male mice maintained on an RCD. *Nampt* mRNA expression in isolated CD31‐positive endothelial cells (*n* = 4–8 per group). Data were analyzed using Student's unpaired *t*‐test. All values are presented as the mean ± SEM. *p < 0.05.

**Figure S9:** **Minimal effect of endothelial NAD^+^** **decline on eNOS activity, glucose metabolism, and cardiovascular homeostasis in aged female mice.** (A–J) C57BL/6 female mice were fed an RCD. Young and aged mice were 2–3 months old (white bar) and 1.5 years old (black bar), respectively. (A) NAD^+^ levels in CD31‐positive vascular endothelial cells isolated from the lungs (*n* = 4–7 per group). (B) Western blotting of NAMPT levels in CD31‐positive vascular endothelial cells isolated from the lungs. Band intensities were quantified and normalized to those of β‐actin (*n* = 4 per group). (C) Western blotting of aortic p‐eNOS levels in young and aged female mice. Band intensities were quantified and normalized to those of native eNOS (*n* = 4–5 per group). (D) Blood glucose concentration in insulin tolerance tests (*n* = 8–10 per group). (E) Fat depot masses. Relative fat masses were normalized to the average fat mass in young mice, which was set as 1 (*n* = 5–6 per group). (F) Representative micrographs of CD31‐positive cells in sWAT. Left: low magnification; right: high magnification. Arrowheads denote CD31‐positive cells (brown, endothelial cell marker). Scale bar, 100 μm. Quantification of CD31‐positive vessel densities as CD31‐positive area/field (*n* = 5 per group). (G) mRNA expression levels of genes involved in angiogenesis in sWAT (*n* = 5–7 per group). (H) SBP and DBP (mmHg) (*n* = 8–10 per group). (I) Cardiac masses (*n* = 4–6 per group). (J) Aortic media thickness/lumen diameter (*n* = 5 per group). Data were analyzed using Student's unpaired *t*‐test. All values are presented as the mean ± SEM. *p < 0.05; **p < 0.01.

**Figure S10:** **NMN administration does not affect body weight gain in aged mice.** NMN (500 mg/kg body weight/day, up to 11 weeks) was added to the drinking water of aged mice (1.5 years old) fed an RCD. Body weights of untreated (black bar) and NMN‐treated (gray bar) aged male mice at 0, 5, and 10 weeks of NMN treatment (*n* = 5 per group). Data were analyzed using Student's unpaired *t*‐test. All values are presented as the mean ± SEM.

**Table S1:** **Cardiometabolic profiles in fl/fl and VeNKO mice of both sexes fed a high‐fat diet.**

**Table S2:** **Cardiovascular markers in young and aged mice of both sexes**

**Table S3:** **Primers used for real‐time PCR.**

## Data Availability

The data that support the findings of this study are available from the corresponding author upon reasonable request.
