# Supplementary figures and images for: Vascular Endothelial NAMPT‐Mediated NAD + Biosynthesis Regulates Angiogenesis and Cardiometabolic Functions in Male Mice
Source: Aging Cell. 2025 Sep 29;24(11):e70222. doi: 10.1111/acel.70222 (PMC12608088; doi:10.1111/acel.70222)

## Slide 1
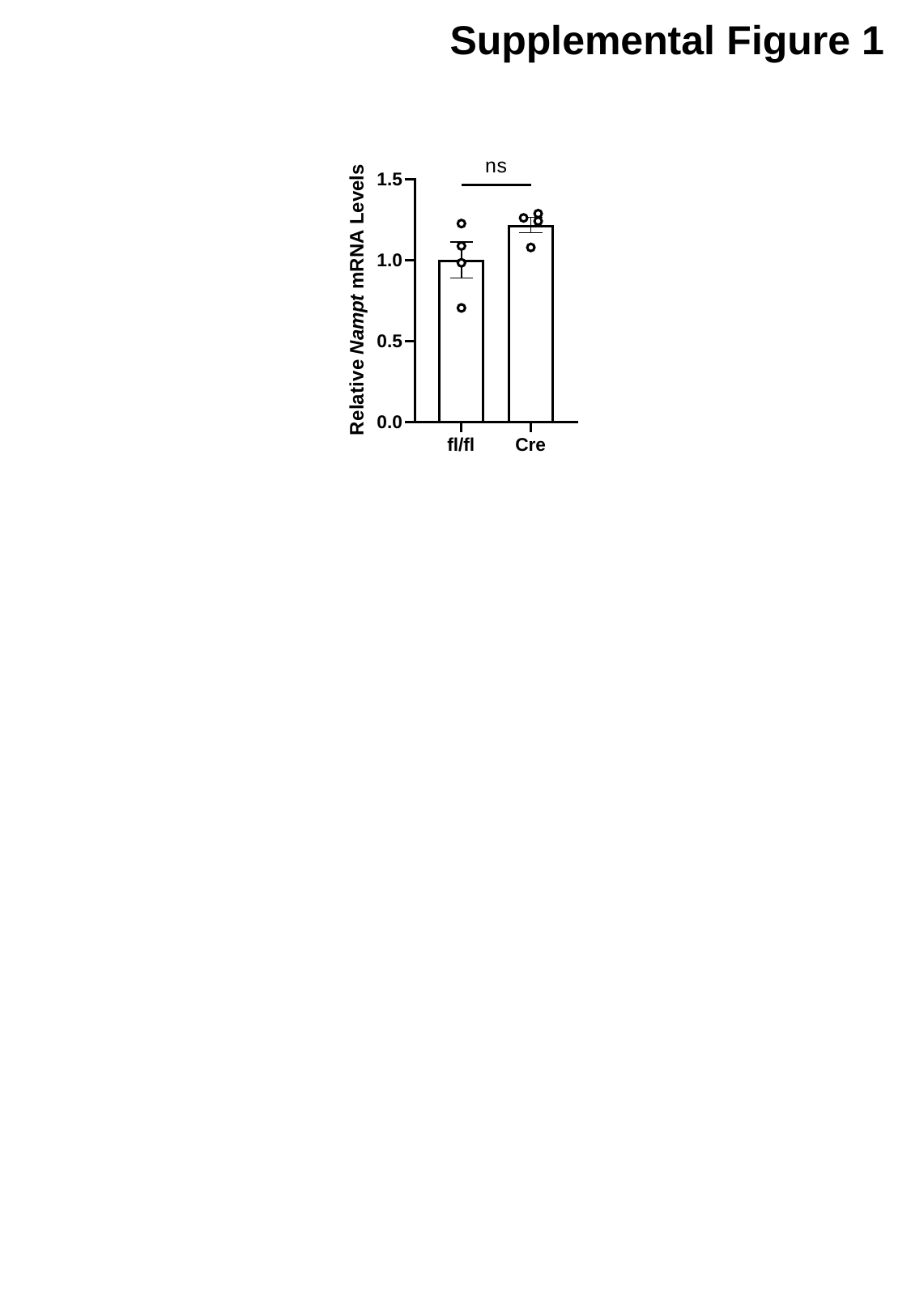

Supplemental Figure 1

Supplement: Supplementary file 1 — Figure S1: Ve‐cadherin‐Cre transgenic mice exhibit Nampt expression levels comparable to those observed in fl/fl mice. Quantification of Nampt expression in CD31‐positive endothelial cells isolated from the visceral white adipose (vWAT) tissue of 2‐month‐old Ve‐cadherin‐Cre transgenic (Cre) and control (fl/fl) male mice (n = 4 per group). Data were analyzed using Student's unpaired t‐test. All data are presented as the mean ± standard error of the mean (SEM). [file ACEL-24-e70222-s005.pptx]

## Slide 1
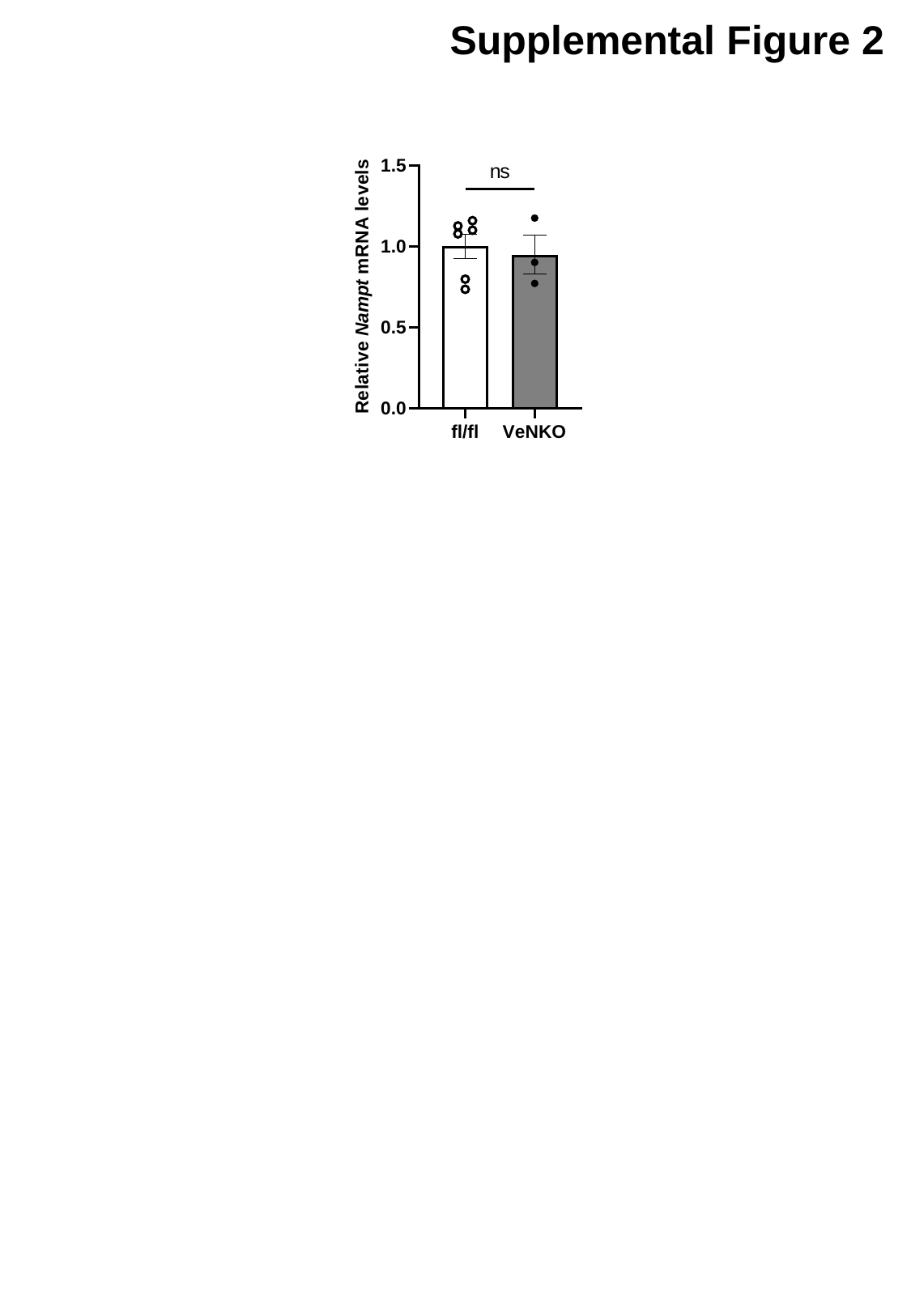

Supplemental Figure 2

Supplement: Supplementary file 2 — Figure S2: VeNKO mice maintain Nampt expression in hematopoietic lineage cells similar to fl/fl mice. Nampt mRNA levels were quantitatively assessed in hematopoietic lineage cells included in total bone marrow cell isolates obtained from 2‐month‐old fl/fl and VeNKO male mice (n = 3–6 per group). Data were analyzed using Student's unpaired t‐test. All data are presented as the mean ± SEM. [file ACEL-24-e70222-s011.pptx]

## Slide 1
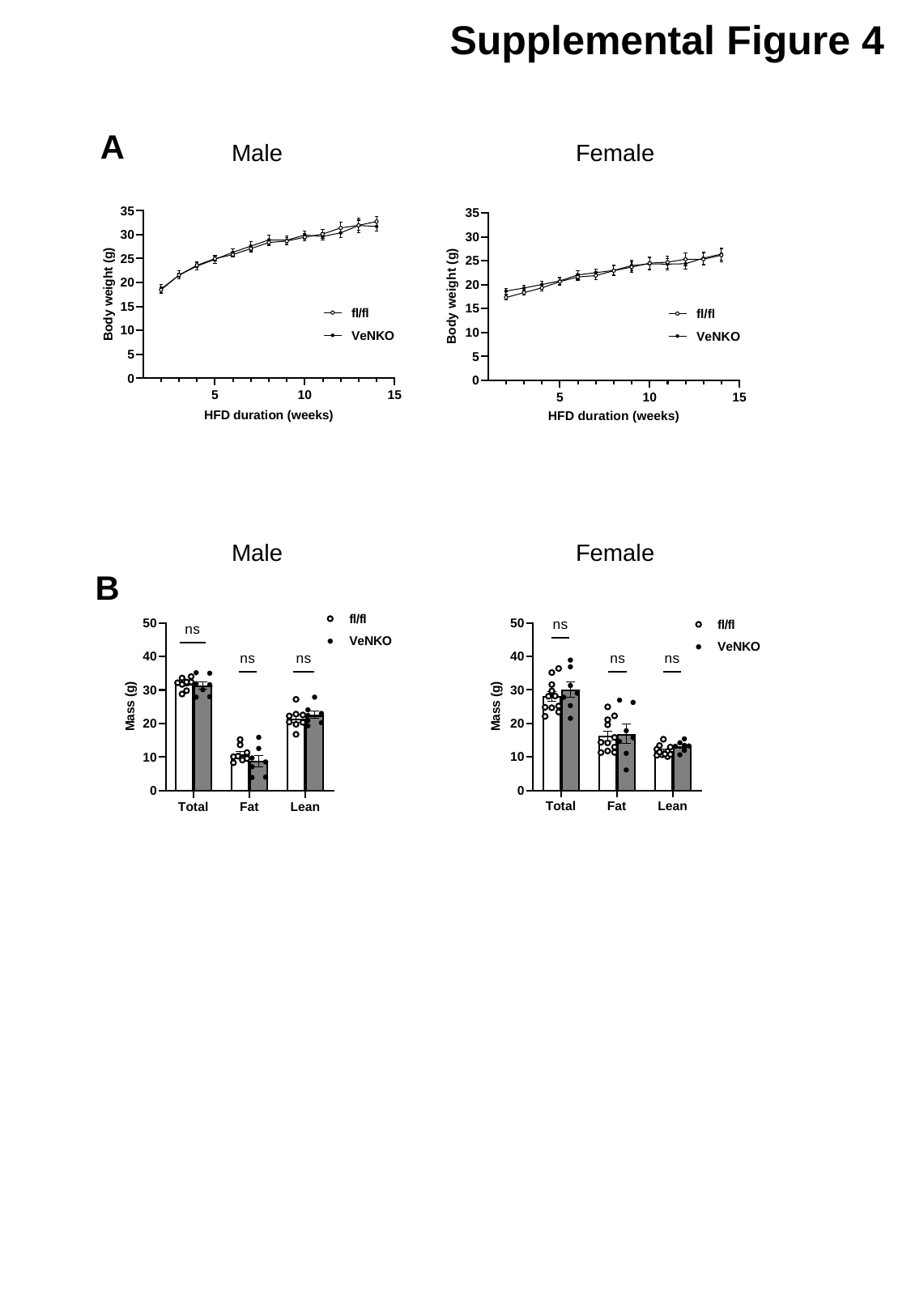

Supplemental Figure 4
A
Male
Female
Male
Female
B

Supplement: Supplementary file 4 — Figure S4: Nampt deficiency in vascular endothelial cells has minimal effect on body weight and composition in mice fed a high‐fat diet (HFD). Both fl/fl and VeNKO mice were fed an HFD. (A) Body weights (n = 7–13 per group) and (B) body composition (n = 7–11 per group) were measured in 4–5‐month‐old VeNKO and fl/fl male and female mice. Data were analyzed using Student's unpaired t‐test. All values are presented as the mean ± SEM. [file ACEL-24-e70222-s004.pptx]

## Slide 1
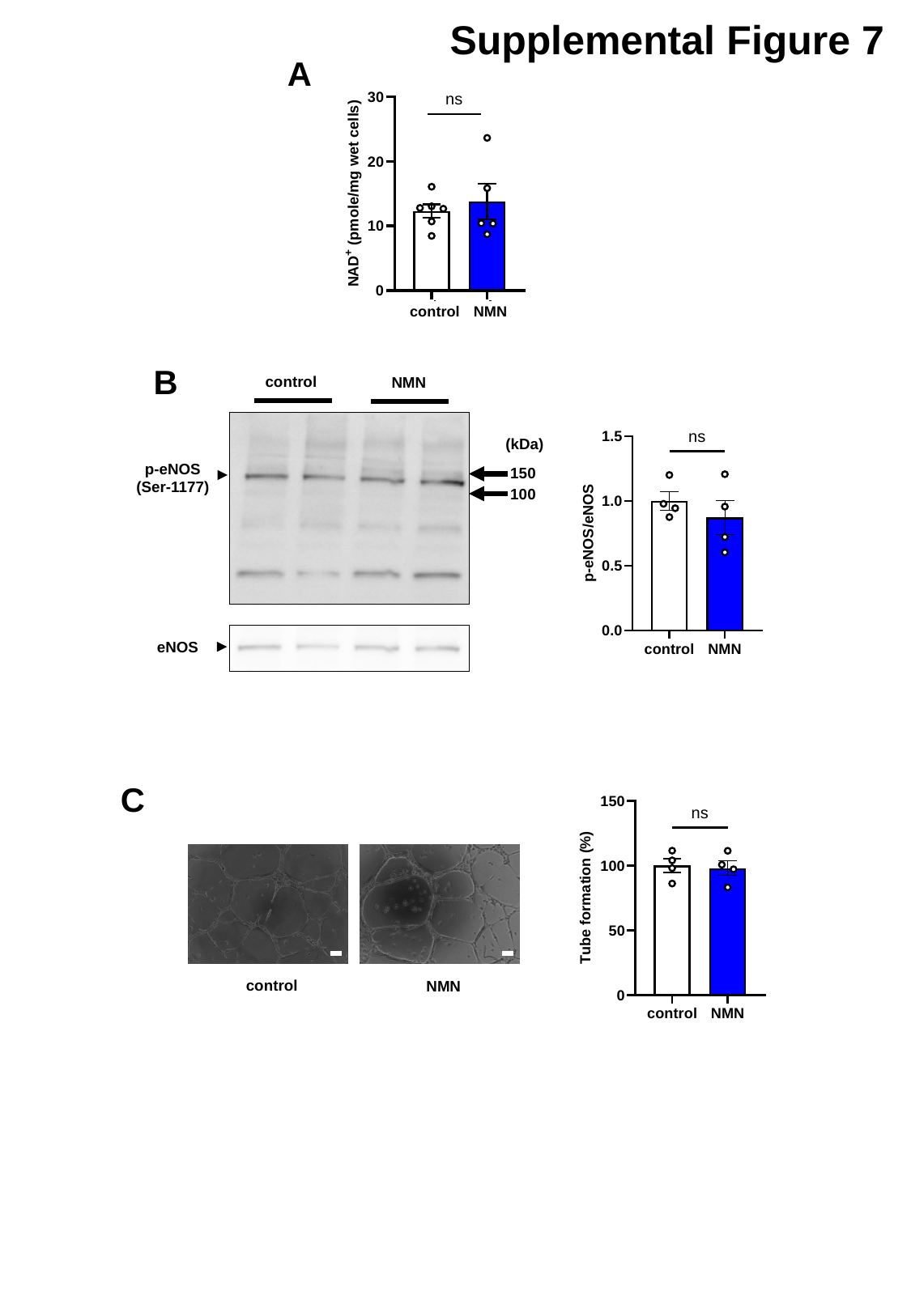

Supplemental Figure 7
A
B
control
NMN
(kDa)
p-eNOS
(Ser-1177)
150
100
eNOS
C
control
NMN

Supplement: Supplementary file 7 — Figure S7: NMN supplementation alone does not enhance eNOS or angiogenic activity in endothelial cells. (A–C) Human umbilical vein endothelial cells (HUVECs) were cultured for 16 h in the presence of either water (vehicle control, white bars) or 10 μM NMN (blue bars). (A) NAD+ concentrations measured following treatment (n = 5–6 per group). (B) Western blot analysis of levels of p‐eNOS in HUVECs (n = 4 per group). Band intensities of p‐eNOS were normalized to total eNOS levels. (C) HUVECs were cultured in Matrigel to generate capillary‐like structures. Representative micrographs of HUVEC tube assays in the presence of either water or NMN for 16 h (scale bar = 200 μm). The branches were counted and averaged. Tube formation under NMN‐treatment was normalized to that of the control group, which was set as 100% (n = 4 per condition). Data were analyzed using Student's unpaired t‐test. All values are presented as the mean ± SEM. [file ACEL-24-e70222-s003.pptx]

## Slide 1
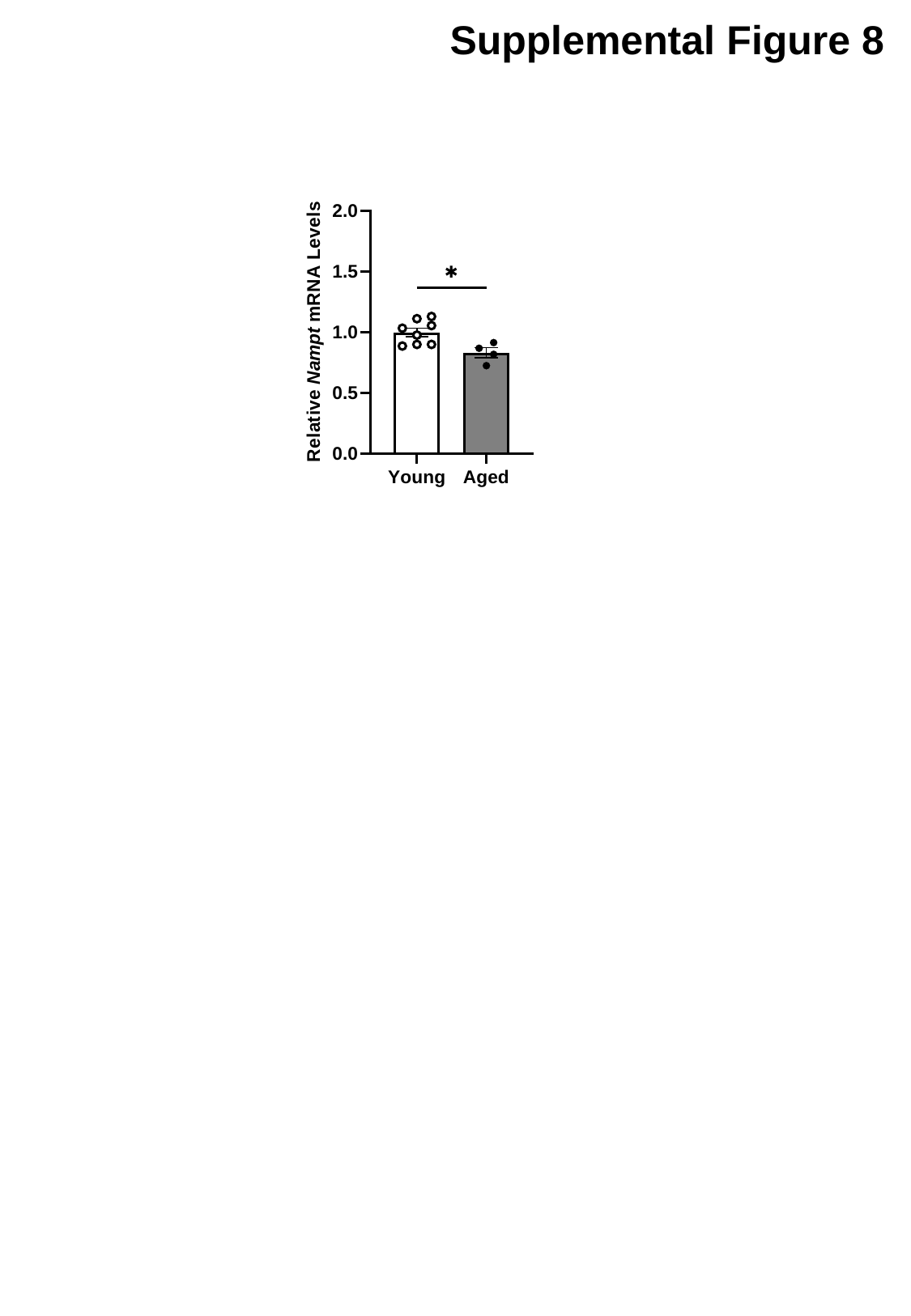

Supplemental Figure 8

Supplement: Supplementary file 8 — Figure S8: Aging reduces Nampt expression in endothelial cells. CD31‐positive endothelial cells were isolated from white adipose tissue of young (2–3 months; white bars) and aged (18–24 months; black bars) C57BL/6 male mice maintained on an RCD. Nampt mRNA expression in isolated CD31‐positive endothelial cells (n = 4–8 per group). Data were analyzed using Student's unpaired t‐test. All values are presented as the mean ± SEM. *p < 0.05. [file ACEL-24-e70222-s009.pptx]

## Slide 1
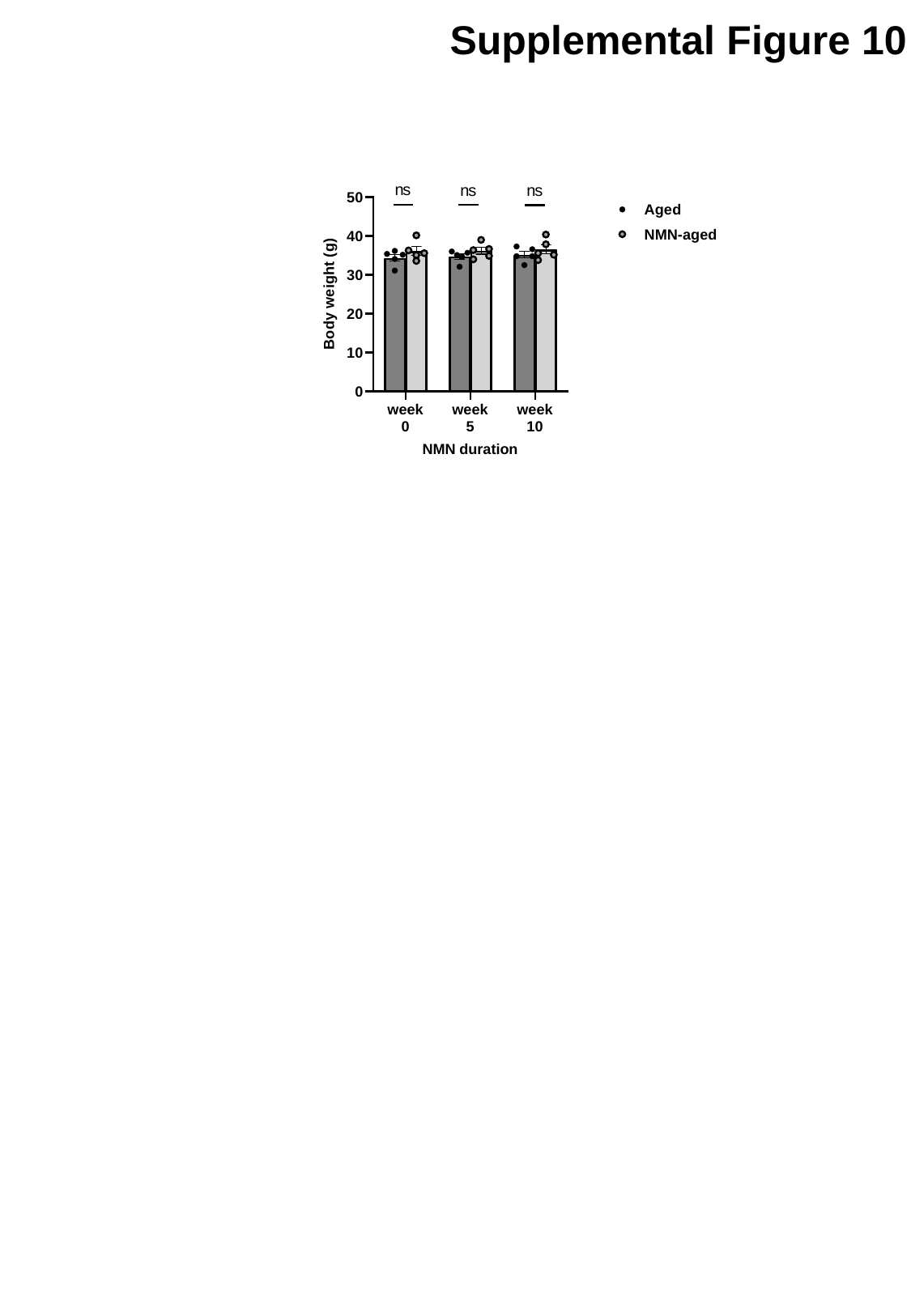

Supplemental Figure 10

Supplement: Supplementary file 10 — Figure S10: NMN administration does not affect body weight gain in aged mice. NMN (500 mg/kg body weight/day, up to 11 weeks) was added to the drinking water of aged mice (1.5 years old) fed an RCD. Body weights of untreated (black bar) and NMN‐treated (gray bar) aged male mice at 0, 5, and 10 weeks of NMN treatment (n = 5 per group). Data were analyzed using Student's unpaired t‐test. All values are presented as the mean ± SEM. [file ACEL-24-e70222-s001.pptx]
