# Supplementary material for: Vascular Endothelial NAMPT‐Mediated NAD + Biosynthesis Regulates Angiogenesis and Cardiometabolic Functions in Male Mice
Source: Aging Cell. 2025 Sep 29;24(11):e70222. doi: 10.1111/acel.70222 (PMC12608088; doi:10.1111/acel.70222)
Supplement: Supplementary file 3 — Figure S3: Vascular endothelial cell‐specific Nampt deletion has minimal effect on energy and glucose metabolism and cardiovascular homeostasis in mice fed a regular chow diet (RCD). Characterization of fl/fl and vascular endothelial cell‐specific Nampt knockout (VeNKO) mice fed an RCD. (A) Body weights of fl/fl and VeNKO mice. Body weights of male (n = 6–10 per group) and female (n = 12–13 per group) mice were monitored soon after weaning. A significant time effect (p < 0.05) was observed without any group × time interaction (ANOVA). (B) Body composition and organ weights: liver, kidneys, heart, subcutaneous white adipose tissue (sWAT), vWAT, and brown adipose tissue (BAT), were measured in 5–6‐month‐old female mice (n = 5 per group). (C) Daily food intake in 2–3‐month‐old female mice (n = 5 per group). (D) Respiratory quotient and energy expenditure in 2–3‐month‐old female mice (n = 4–5 per group). Blood glucose concentrations measured using (E) glucose (n = 5–16 per group) and (F) insulin (n = 4–8 per group) tolerance tests in 2–5‐month‐old male and female mice. The area under the curve (AUC) for glucose during the glucose tolerance test is displayed alongside the glucose curves for both males and females. (G) Systolic blood pressure (SBP) (mmHg) in 3–5‐month‐old male and female mice (n = 4–6 per group). (H) Cardiac masses in 5–6‐month‐old female mice (n = 5 per group). Data were analyzed using Student's unpaired t‐test. All values are presented as the mean ± SEM. [file ACEL-24-e70222-s002.pptx]

## Slide 1
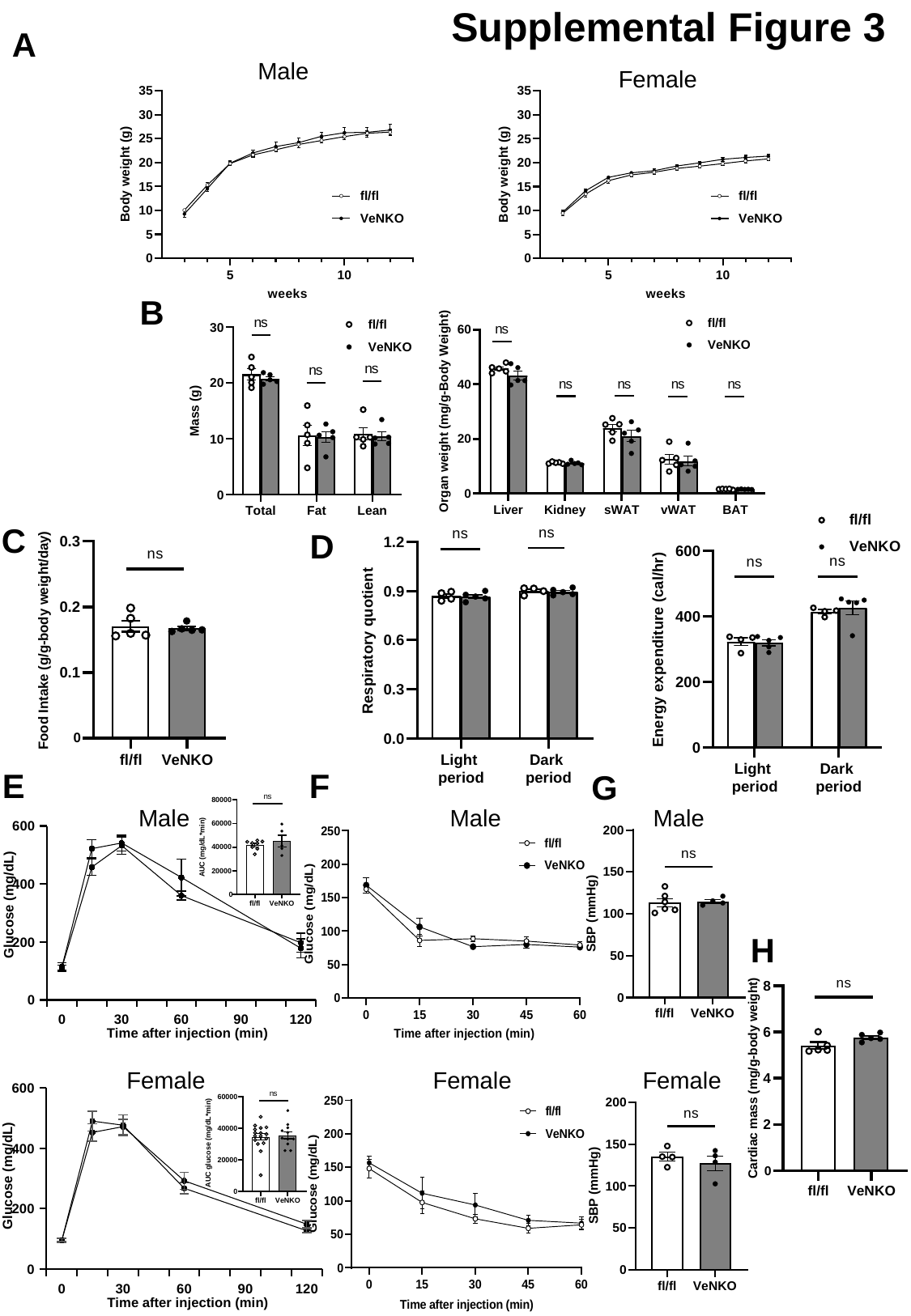

Supplemental Figure 3
A
Male
Female
B
C
D
E
F
G
Male
Male
Male
### Chart
| Category | f/f | VeNKO |
|---|---|---|
| 0 | 111.625 | 113.8 |
| | 458.0 | 521.8 |
| 30 | 532.25 | 541.2 |
| | None | None |
| 60 | 359.75 | 422.4 |
| | None | None |
| 90 | None | None |
| | None | None |
| 120 | 197.25 | 178.2 |Glucose (mg/dL)
H
Time after injection (min)
Female
Female
Female
### Chart
| Category | f/f | VeNKO |
|---|---|---|
| 0 | 95.125 | 95.27272727272727 |
| | 489.8125 | 452.27272727272725 |
| 30 | 476.125 | 471.45454545454544 |
| | None | None |
| 60 | 267.5 | 292.1818181818182 |
| | None | None |
| 90 | None | None |
| | None | None |
| 120 | 126.8125 | 148.45454545454547 |Glucose (mg/dL)
Time after injection (min)
