# Supplementary material for: Vascular Endothelial NAMPT‐Mediated NAD + Biosynthesis Regulates Angiogenesis and Cardiometabolic Functions in Male Mice
Source: Aging Cell. 2025 Sep 29;24(11):e70222. doi: 10.1111/acel.70222 (PMC12608088; doi:10.1111/acel.70222)
Supplement: Supplementary file 5 — Figure S5: Vascular endothelial NAMPT deficiency affects insulin signaling in sWAT, without impairing liver or skeletal muscle metabolism in HFD‐fed VeNKO mice. Western blot analysis of levels of phosphorylated Akt at Ser473 (p‐Akt) in the (A) liver (n = 4–5 per group), (C) skeletal muscle (n = 4–5 per group), and (E) sWAT (n = 4–5 per group) after 15–19 weeks of HFD feeding. Band intensities were quantified and normalized to total Akt levels. Triglyceride levels in the (B) liver (n = 5 per group), (D) skeletal muscle (n = 6–7 per group), and (F) sWAT (n = 4 per group) were measured after 14–17 weeks of HFD feeding. Data were analyzed using unpaired Student's t‐test. Values are presented as the mean ± SEM. *p < 0.05. [file ACEL-24-e70222-s007.pptx]

## Slide 1
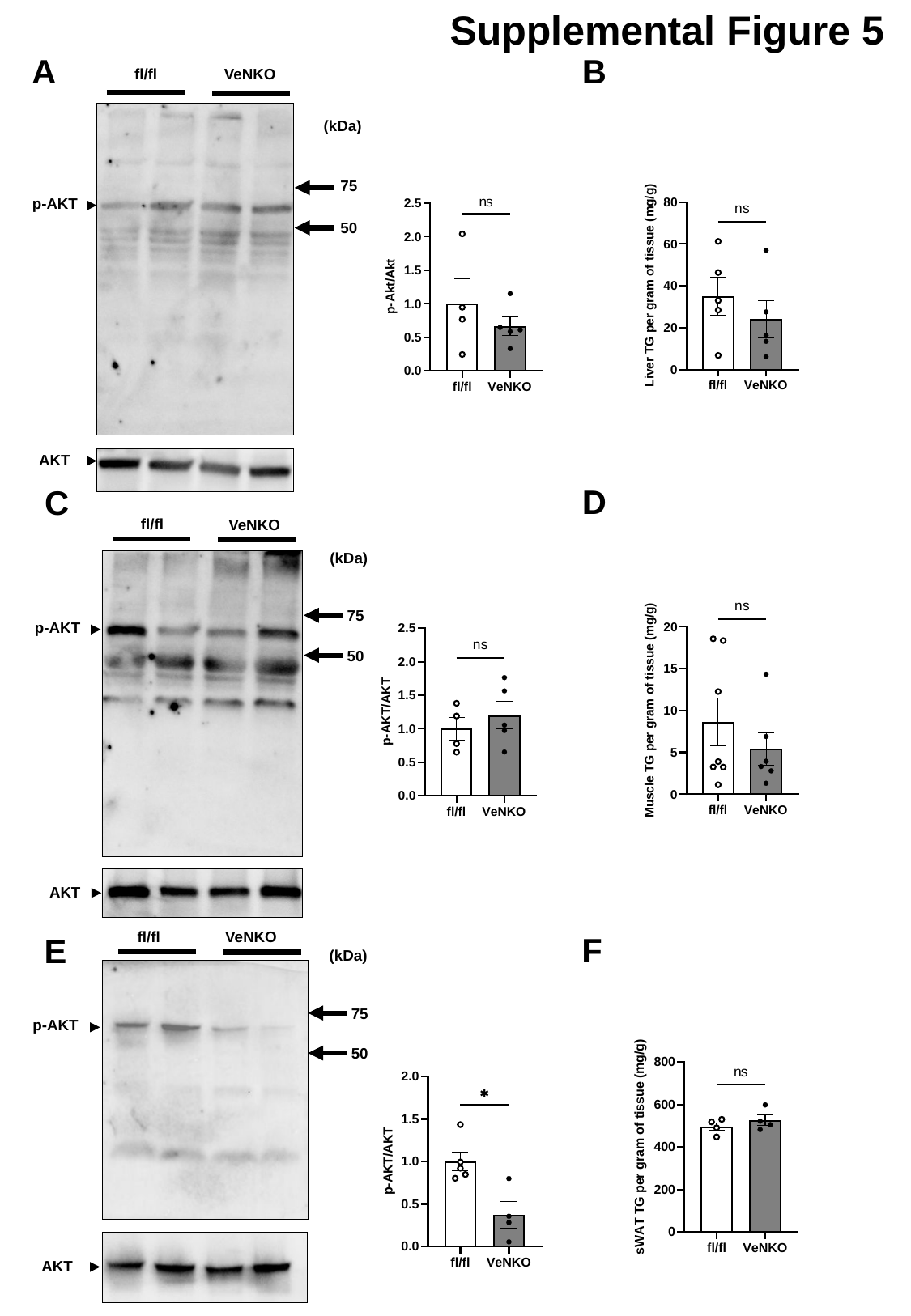

Supplemental Figure 5
B
A
fl/fl
VeNKO
(kDa)
75
p-AKT
50
AKT
D
C
fl/fl
VeNKO
(kDa)
75
p-AKT
50
AKT
fl/fl
VeNKO
F
E
(kDa)
75
p-AKT
50
AKT
