# Supplementary material for: Vascular Endothelial NAMPT‐Mediated NAD + Biosynthesis Regulates Angiogenesis and Cardiometabolic Functions in Male Mice
Source: Aging Cell. 2025 Sep 29;24(11):e70222. doi: 10.1111/acel.70222 (PMC12608088; doi:10.1111/acel.70222)
Supplement: Supplementary file 6 — Figure S6: Vascular endothelial cell‐specific Nampt deletion in female mice fed a HFD has minimal effect on glucose metabolism and cardiovascular homeostasis. (A) NAD+ levels in endothelial cells isolated from the lungs of mice after 16–19 weeks of HFD (n = 5–8 per group). (B) Western blotting of levels of phosphorylated endothelial nitric oxide synthase (eNOS) at Ser‐1177 (p‐eNOS) in the aortas of female VeNKO and fl/fl mice fed HFD for 16–19 weeks. Band intensities were quantified and normalized to those of native eNOS (n = 3–5 per group). (C) Glucose (n = 7–11 per group) and (E) insulin (n = 6–11 per group) tolerance test results performed after 10–13 weeks of HFD. The AUC for glucose during the glucose tolerance test is displayed next to the glucose curves. (D) Plasma insulin concentrations during the glucose tolerance tests following overnight fasting (n = 4–5 per group). (F) Evaluation of fat depot masses, including sWAT and vWAT, after 16–19 weeks of HFD feeding. Relative fat mass was normalized to that of the fl/fl group, which was set as 1 (n = 7–9 per group). (G) Representative micrographs of immunohistochemical staining for CD31 in sWAT after 16–19 weeks of HFD feeding (n = 5 per group). Left: low magnification; right: high magnification. Arrowheads denote CD31‐positive cells (brown, endothelial cell marker). Scale bar, 100 μm. Quantification of CD31‐positive areas per field per mouse (n = 5 per group). (H) mRNA expression levels of genes involved in angiogenesis in sWAT after 15–19 weeks of HFD feeding (n = 5–7 per group). (I) SBP and diastolic blood pressure (DBP) (mmHg) measured after 15–16 weeks of HFD (n = 7–8 per group). (J) Cardiac masses after 16–19 weeks of HFD (n = 7–11 per group). (K) Aortic media thickness/lumen diameter (n = 5 per group). Data were analyzed using Student's unpaired t‐test. All values are presented as the mean ± SEM. **p < 0.01. [file ACEL-24-e70222-s010.pptx]

## Slide 1
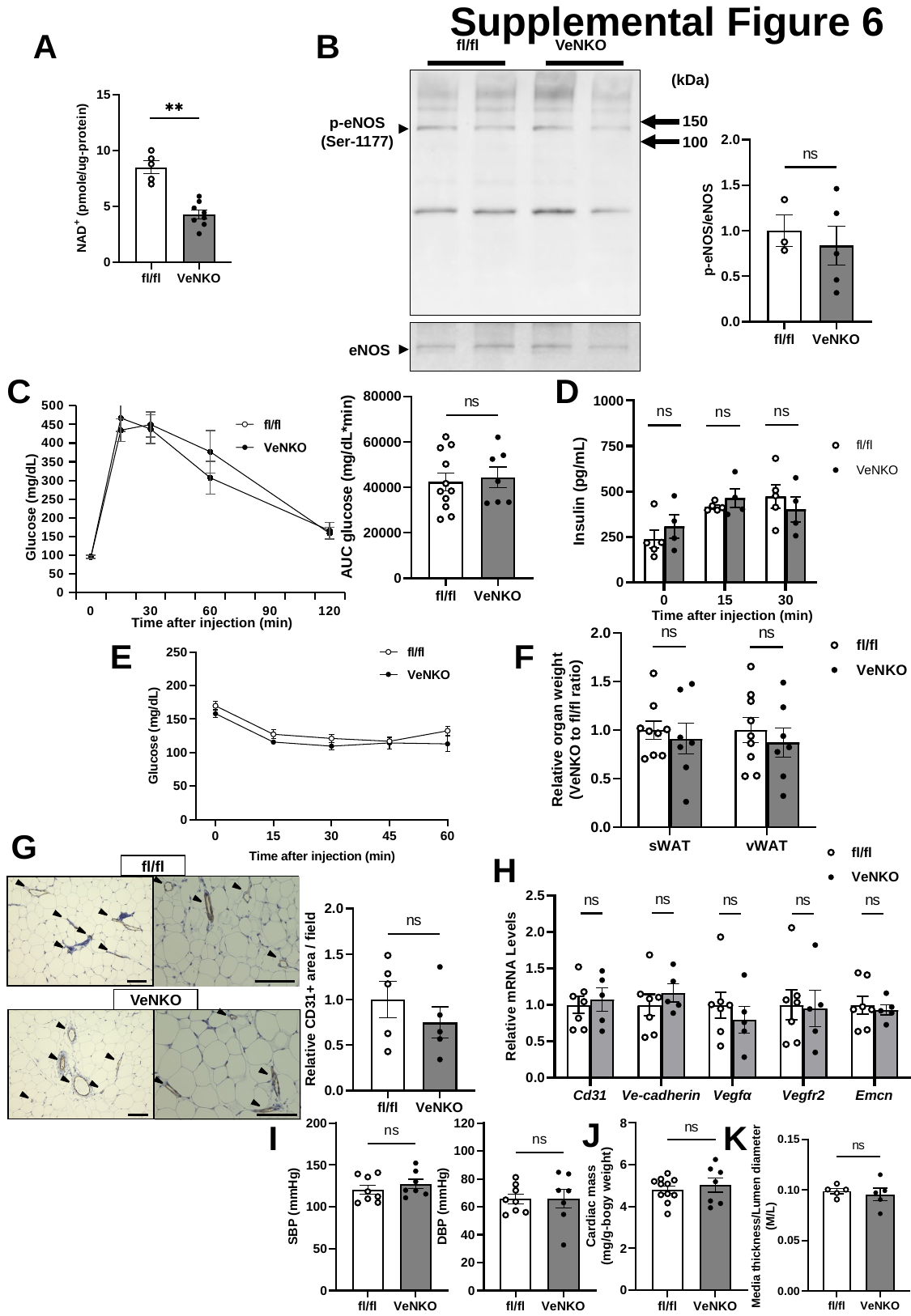

Supplemental Figure 6
A
B
fl/fl
VeNKO
(kDa)
150
p-eNOS
(Ser-1177)
100
eNOS
C
D
### Chart
| Category | fl/fl | VeNKO |
|---|---|---|
| 0 | 95.8 | 94.8 |
| | 466.9 | 434.6 |
| 30 | 437.55 | 449.7 |
| | None | None |
| 60 | 307.0 | 376.8 |
| | None | None |
| 90 | None | None |
| | None | None |
| 120 | 165.3 | 158.2 |
Glucose (mg/dL)
Time after injection (min)
Time after injection (min)
E
F
G
H
fl/fl
VeNKO
J
I
K
Media thickness/Lumen diameter
(M/L)
