# Supplementary material for: Vascular Endothelial NAMPT‐Mediated NAD + Biosynthesis Regulates Angiogenesis and Cardiometabolic Functions in Male Mice
Source: Aging Cell. 2025 Sep 29;24(11):e70222. doi: 10.1111/acel.70222 (PMC12608088; doi:10.1111/acel.70222)
Supplement: Supplementary file 9 — Figure S9: Minimal effect of endothelial NAD+ decline on eNOS activity, glucose metabolism, and cardiovascular homeostasis in aged female mice. (A–J) C57BL/6 female mice were fed an RCD. Young and aged mice were 2–3 months old (white bar) and 1.5 years old (black bar), respectively. (A) NAD+ levels in CD31‐positive vascular endothelial cells isolated from the lungs (n = 4–7 per group). (B) Western blotting of NAMPT levels in CD31‐positive vascular endothelial cells isolated from the lungs. Band intensities were quantified and normalized to those of β‐actin (n = 4 per group). (C) Western blotting of aortic p‐eNOS levels in young and aged female mice. Band intensities were quantified and normalized to those of native eNOS (n = 4–5 per group). (D) Blood glucose concentration in insulin tolerance tests (n = 8–10 per group). (E) Fat depot masses. Relative fat masses were normalized to the average fat mass in young mice, which was set as 1 (n = 5–6 per group). (F) Representative micrographs of CD31‐positive cells in sWAT. Left: low magnification; right: high magnification. Arrowheads denote CD31‐positive cells (brown, endothelial cell marker). Scale bar, 100 μm. Quantification of CD31‐positive vessel densities as CD31‐positive area/field (n = 5 per group). (G) mRNA expression levels of genes involved in angiogenesis in sWAT (n = 5–7 per group). (H) SBP and DBP (mmHg) (n = 8–10 per group). (I) Cardiac masses (n = 4–6 per group). (J) Aortic media thickness/lumen diameter (n = 5 per group). Data were analyzed using Student's unpaired t‐test. All values are presented as the mean ± SEM. *p < 0.05; **p < 0.01. [file ACEL-24-e70222-s008.pptx]

## Slide 1
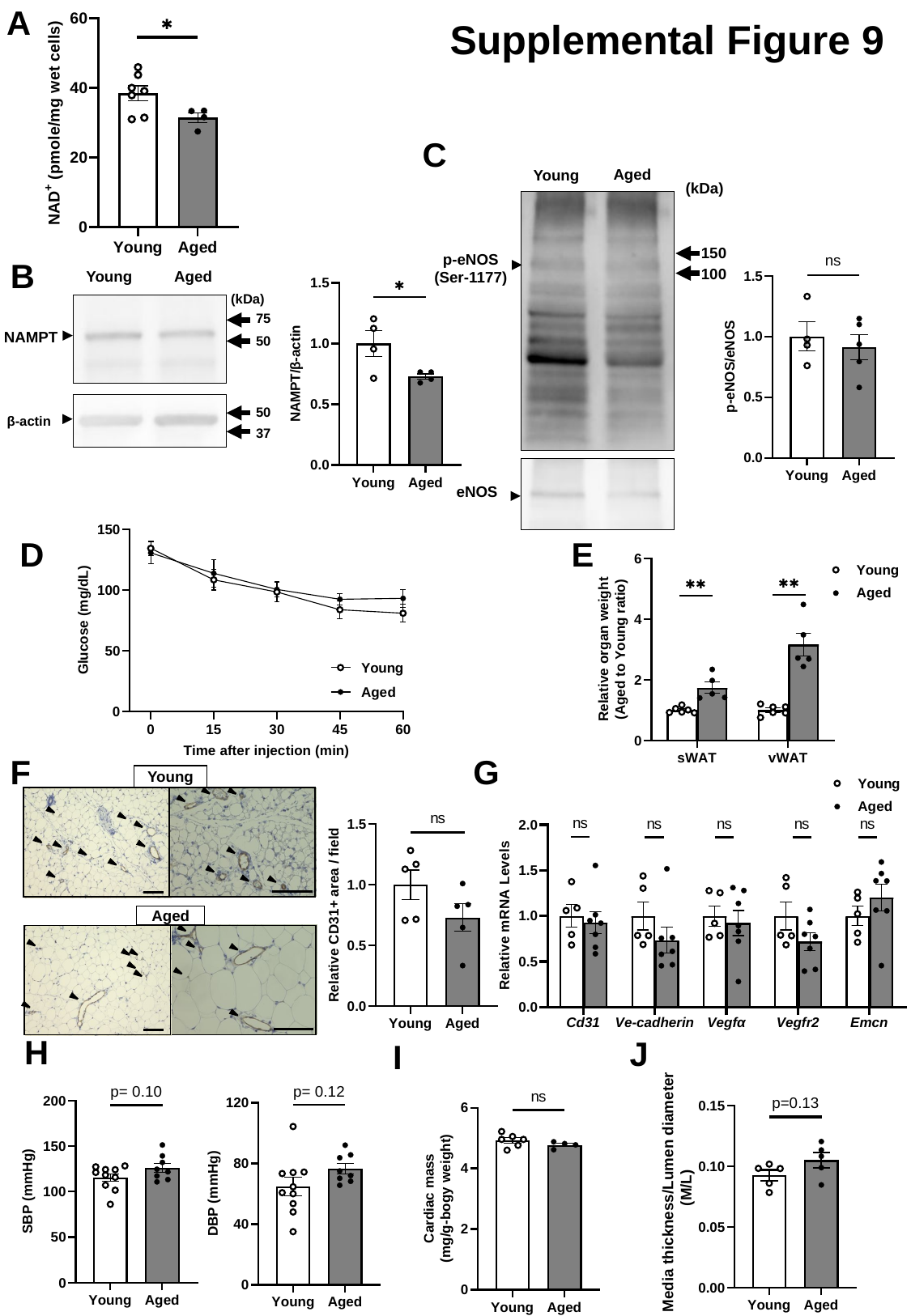

A
Supplemental Figure 9
C
Aged
Young
(kDa)
150
p-eNOS
(Ser-1177)
B
100
Young Aged
(kDa)
75
NAMPT
50
50
β-actin
37
eNOS
D
E
F
G
Young
Aged
H
J
I
Media thickness/Lumen diameter
 (M/L)
