# Supplementary material for: Vascular Endothelial NAMPT‐Mediated NAD + Biosynthesis Regulates Angiogenesis and Cardiometabolic Functions in Male Mice
Source: Aging Cell. 2025 Sep 29;24(11):e70222. doi: 10.1111/acel.70222 (PMC12608088; doi:10.1111/acel.70222)
Supplement: Supplementary file 11 — Table S1: Cardiometabolic profiles in fl/fl and VeNKO mice of both sexes fed a high‐fat diet. [file ACEL-24-e70222-s013.pptx]

## Slide 1
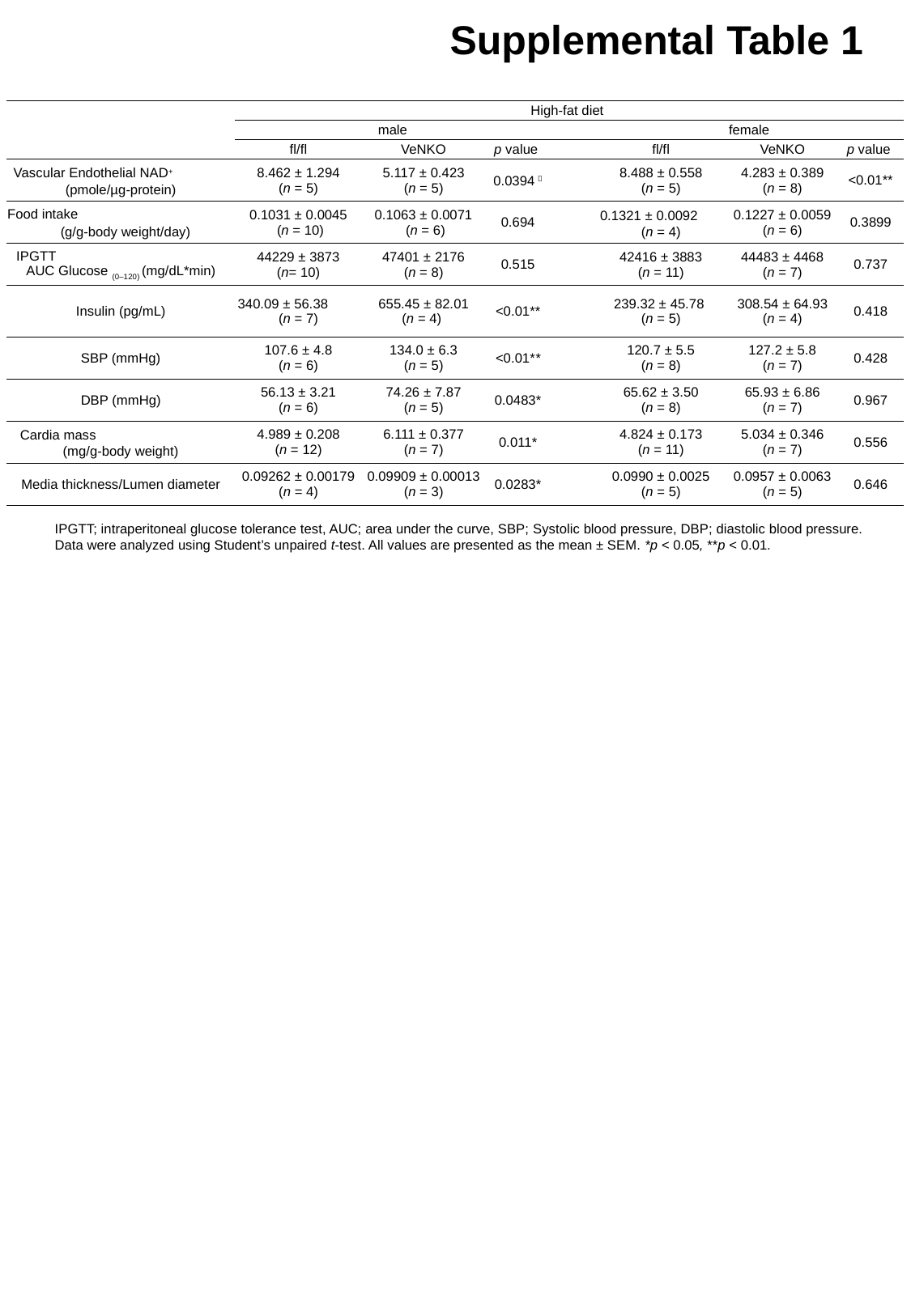

Supplemental Table 1
| | High-fat diet | | | | | | |
| --- | --- | --- | --- | --- | --- | --- | --- |
| | male | | | | female | | |
| | fl/fl | VeNKO | p value | | fl/fl | VeNKO | p value |
| Vascular Endothelial NAD+ 　 　(pmole/µg-protein) | 8.462 ± 1.294 (n = 5) | 5.117 ± 0.423 (n = 5) | 0.0394＊ | | 8.488 ± 0.558 (n = 5) | 4.283 ± 0.389 (n = 8) | <0.01\*\* |
| Food intake 　　　　　　　　　　　　(g/g-body weight/day) | 0.1031 ± 0.0045 (n = 10) | 0.1063 ± 0.0071 (n = 6) | 0.694 | | 0.1321 ± 0.0092 　(n = 4) | 0.1227 ± 0.0059 (n = 6) | 0.3899 |
| IPGTT AUC Glucose (0–120) (mg/dL\*min) | 44229 ± 3873 (n= 10) | 47401 ± 2176 (n = 8) | 0.515 | | 42416 ± 3883 (n = 11) | 44483 ± 4468 (n = 7) | 0.737 |
| Insulin (pg/mL) | 340.09 ± 56.38 (n = 7) | 655.45 ± 82.01 (n = 4) | <0.01\*\* | | 239.32 ± 45.78 (n = 5) | 308.54 ± 64.93 (n = 4) | 0.418 |
| SBP (mmHg) | 107.6 ± 4.8 (n = 6) | 134.0 ± 6.3 (n = 5) | <0.01\*\* | | 120.7 ± 5.5 (n = 8) | 127.2 ± 5.8 (n = 7) | 0.428 |
| DBP (mmHg) | 56.13 ± 3.21 (n = 6) | 74.26 ± 7.87 (n = 5) | 0.0483\* | | 65.62 ± 3.50 (n = 8) | 65.93 ± 6.86 (n = 7) | 0.967 |
| Cardia mass　　　　　　　　　 (mg/g-body weight) | 4.989 ± 0.208 (n = 12) | 6.111 ± 0.377 (n = 7) | 0.011\* | | 4.824 ± 0.173 (n = 11) | 5.034 ± 0.346 (n = 7) | 0.556 |
| Media thickness/Lumen diameter | 0.09262 ± 0.00179 (n = 4) | 0.09909 ± 0.00013 (n = 3) | 0.0283\* | | 0.0990 ± 0.0025 (n = 5) | 0.0957 ± 0.0063 (n = 5) | 0.646 |
IPGTT; intraperitoneal glucose tolerance test, AUC; area under the curve, SBP; Systolic blood pressure, DBP; diastolic blood pressure.
Data were analyzed using Student’s unpaired t-test. All values are presented as the mean ± SEM. *p < 0.05, **p < 0.01.
