# Supplementary material for: Vascular Endothelial NAMPT‐Mediated NAD + Biosynthesis Regulates Angiogenesis and Cardiometabolic Functions in Male Mice
Source: Aging Cell. 2025 Sep 29;24(11):e70222. doi: 10.1111/acel.70222 (PMC12608088; doi:10.1111/acel.70222)
Supplement: Supplementary file 12 — Table S2: Cardiovascular markers in young and aged mice of both sexes [file ACEL-24-e70222-s006.pptx]

## Slide 1
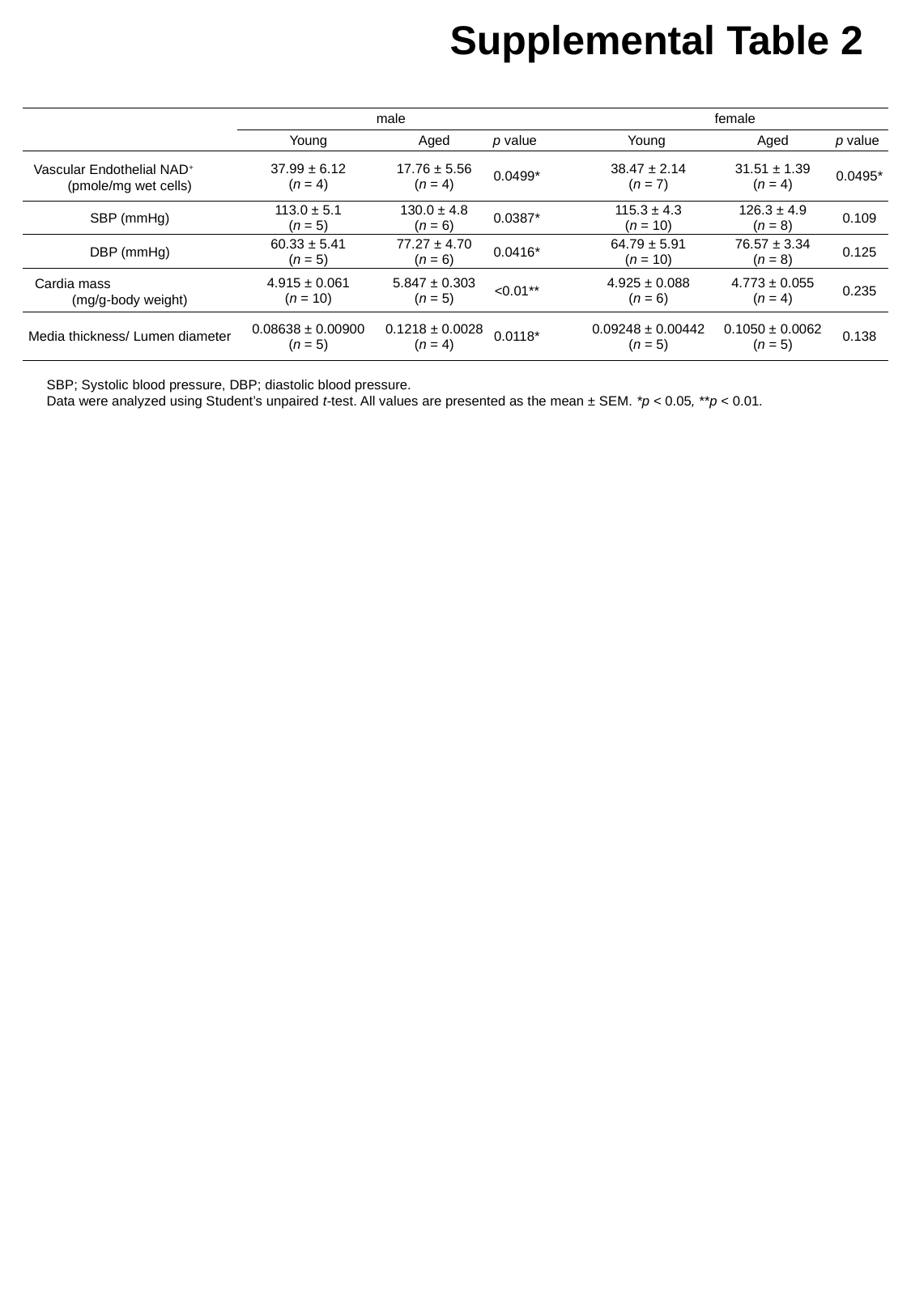

Supplemental Table 2
| | male | | | | female | | |
| --- | --- | --- | --- | --- | --- | --- | --- |
| | Young | Aged | p value | | Young | Aged | p value |
| Vascular Endothelial NAD+　　(pmole/mg wet cells) | 37.99 ± 6.12 (n = 4) | 17.76 ± 5.56 (n = 4) | 0.0499\* | | 38.47 ± 2.14 (n = 7) | 31.51 ± 1.39 (n = 4) | 0.0495\* |
| SBP (mmHg) | 113.0 ± 5.1 (n = 5) | 130.0 ± 4.8 (n = 6) | 0.0387\* | | 115.3 ± 4.3 (n = 10) | 126.3 ± 4.9 (n = 8) | 0.109 |
| DBP (mmHg) | 60.33 ± 5.41 (n = 5) | 77.27 ± 4.70 (n = 6) | 0.0416\* | | 64.79 ± 5.91 (n = 10) | 76.57 ± 3.34 (n = 8) | 0.125 |
| Cardia mass 　　　　 (mg/g-body weight) | 4.915 ± 0.061 (n = 10) | 5.847 ± 0.303 (n = 5) | <0.01\*\* | | 4.925 ± 0.088 (n = 6) | 4.773 ± 0.055 (n = 4) | 0.235 |
| Media thickness/ Lumen diameter | 0.08638 ± 0.00900 (n = 5) | 0.1218 ± 0.0028 (n = 4) | 0.0118\* | | 0.09248 ± 0.00442 (n = 5) | 0.1050 ± 0.0062 (n = 5) | 0.138 |
SBP; Systolic blood pressure, DBP; diastolic blood pressure.
Data were analyzed using Student’s unpaired t-test. All values are presented as the mean ± SEM. *p < 0.05, **p < 0.01.
