# Supplementary material for: Vascular Endothelial NAMPT‐Mediated NAD + Biosynthesis Regulates Angiogenesis and Cardiometabolic Functions in Male Mice
Source: Aging Cell. 2025 Sep 29;24(11):e70222. doi: 10.1111/acel.70222 (PMC12608088; doi:10.1111/acel.70222)
Supplement: Supplementary file 13 — Table S3: Primers used for real‐time PCR. [file ACEL-24-e70222-s012.pptx]

## Slide 1
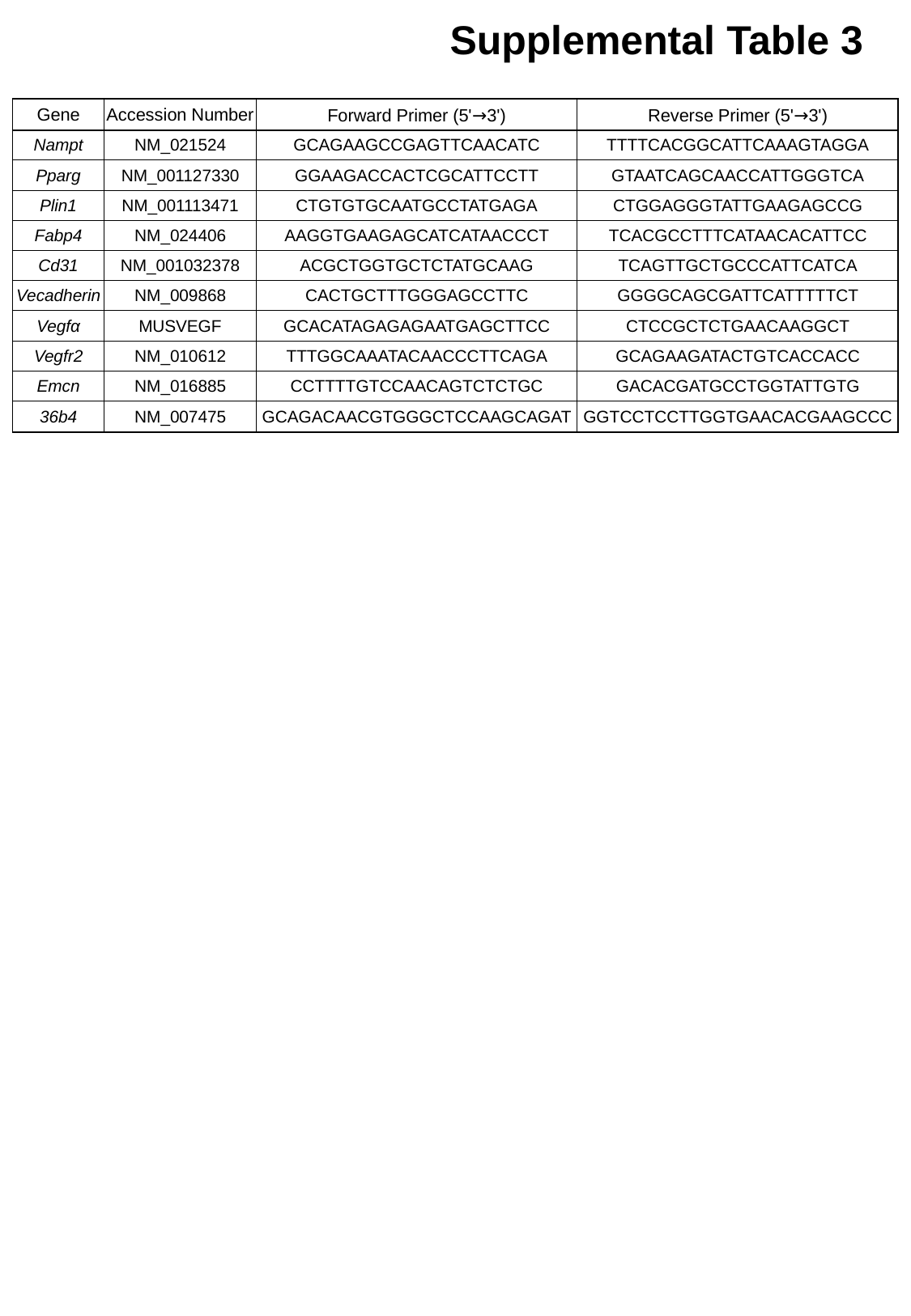

Supplemental Table 3
| Gene | Accession Number | Forward Primer (5'→3') | Reverse Primer (5'→3') |
| --- | --- | --- | --- |
| Nampt | NM\_021524 | GCAGAAGCCGAGTTCAACATC | TTTTCACGGCATTCAAAGTAGGA |
| Pparg | NM\_001127330 | GGAAGACCACTCGCATTCCTT | GTAATCAGCAACCATTGGGTCA |
| Plin1 | NM\_001113471 | CTGTGTGCAATGCCTATGAGA | CTGGAGGGTATTGAAGAGCCG |
| Fabp4 | NM\_024406 | AAGGTGAAGAGCATCATAACCCT | TCACGCCTTTCATAACACATTCC |
| Cd31 | NM\_001032378 | ACGCTGGTGCTCTATGCAAG | TCAGTTGCTGCCCATTCATCA |
| Vecadherin | NM\_009868 | CACTGCTTTGGGAGCCTTC | GGGGCAGCGATTCATTTTTCT |
| Vegfα | MUSVEGF | GCACATAGAGAGAATGAGCTTCC | CTCCGCTCTGAACAAGGCT |
| Vegfr2 | NM\_010612 | TTTGGCAAATACAACCCTTCAGA | GCAGAAGATACTGTCACCACC |
| Emcn | NM\_016885 | CCTTTTGTCCAACAGTCTCTGC | GACACGATGCCTGGTATTGTG |
| 36b4 | NM\_007475 | GCAGACAACGTGGGCTCCAAGCAGAT | GGTCCTCCTTGGTGAACACGAAGCCC |
